# Stochastic nuclear organization and host-dependent allele contribution in Rhizophagus irregularis

**DOI:** 10.1186/s12864-023-09126-6

**Published:** 2023-01-28

**Authors:** Jelle van Creij, Ben Auxier, Jianyong An, Raúl Y. Wijfjes, Claudia Bergin, Anna Rosling, Ton Bisseling, Zhiyong Pan, Erik Limpens

**Affiliations:** 1 Laboratory of Molecular Biology, Department of Plant Sciences, Wageningen University & Research, Droevendaalsesteeg 1, Wageningen, The Netherlands; 2 Laboratory of Genetics, Department of Plant Sciences, Wageningen University & Research, Droevendaalsesteeg 1, Wageningen, The Netherlands; 3 Beijing Advanced Innovation Center for Tree Breeding by Molecular Design, Beijing University of Agriculture, Beijing, 102206 China; 4 Laboratory of Bioinformatics, Department of Plant Sciences, Wageningen University & Research, Droevendaalsesteeg 1, Wageningen, The Netherlands; 5 Current affiliation: Faculty of Biology, Ludwig Maximilian University of Munich, Munich, Germany; 6 Department of Cell and Molecular Biology, Uppsala University, and Microbial Single Cell Genomics Facility, Science for Life Laboratory, Uppsala, Sweden; 7 Department of Ecology and Genetics, Uppsala University, Norbyvägen 18D, SE-75236 Uppsala, Sweden; 8 Key Laboratory of Horticultural Plant Biology (Ministry of Education), Key Laboratory of Horticultural Crop Biology and Genetic Improvement (Central Region, Ministry of Agriculture), College of Horticulture and Forestry Sciences, Huazhong Agricultural University, Wuhan, People’s Republic of China

**Keywords:** Arbuscular mycorrhiza, Heterokaryote, Recombination, Parasexual, Single nucleus sequencing, Symbiosis, *Rhizophagus irregularis*, PacBio SMRT sequencing

## Abstract

**Background:**

Arbuscular mycorrhizal (AM) fungi are arguably the most important symbionts of plants, offering a range of benefits to their hosts. However, the provisioning of these benefits does not appear to be uniform among AM fungal individuals, with genetic variation between fungal symbionts having a substantial impact on plant performance. Interestingly, genetic variation has also been reported within fungal individuals, which contain millions of haploid nuclei sharing a common cytoplasm. In the model AM fungus, *Rhizophagus irregularis*, several isolates have been reported to be dikaryotes, containing two genetically distinct types of nuclei recognized based on their mating-type (MAT) locus identity. However, their extremely coenocytic nature and lack of a known single nucleus stage has raised questions on the origin, distribution and dynamics of this genetic variation.

**Results:**

Here we performed DNA and RNA sequencing at the mycelial individual, single spore and single nucleus levels to gain insight into the dynamic genetic make-up of the dikaryote-like *R. irregularis* C3 isolate and the effect of different host plants on its genetic variation. Our analyses reveal that parallel spore and root culture batches can have widely variable ratios of two main genotypes in C3. Additionally, numerous polymorphisms were found with frequencies that deviated significantly from the general genotype ratio, indicating a diverse population of slightly different nucleotypes. Changing host plants did not show consistent host effects on nucleotype ratio’s after multiple rounds of subculturing. Instead, we found a major effect of host plant-identity on allele-specific expression in C3.

**Conclusion:**

Our analyses indicate a highly dynamic/variable genetic organization in different isolates of *R. irregularis*. Seemingly random fluctuations in nucleotype ratio’s upon spore formation, recombination events, high variability of non-tandemly repeated rDNA sequences and host-dependent allele expression all add levels of variation that may contribute to the evolutionary success of these widespread symbionts.

**Supplementary Information:**

The online version contains supplementary material available at 10.1186/s12864-023-09126-6.

## Background

Fungi belonging to the Glomeromycotina subphylum of the Mucoromycota are globally distributed soil fungi that form an endosymbiosis with the vast majority of land plants [1]. These so-called arbuscular mycorrhizal (AM) fungi rely on their interaction with plants to complete their life cycle. During colonization of plant roots, they form highly branched structures called arbuscules inside root inner cortex cells, where mineral nutrients such as phosphate and nitrogen are exchanged for sugars and fatty acids from the plant [2]. This symbiosis originated more than 400 million years ago and has since been maintained in the vast majority of plants, highlighting its importance in natural ecosystems [3]. Currently, around 315 AM fungal species have been described, however the species concept for these enigmatic fungi is not well defined [4]. Significant intraspecific genetic variation has been observed but evidence for sexual reproduction remains elusive. How the genetic organization of these important fungi contributes to the evolutionary success of this key symbiosis is an important and highly debated question [5, 6]. Large variations in the symbiotic performance, often referred to as mycorrhizal growth response, of different isolates or even among strains derived from single spores from a fungal individual has been reported [7–10]. What determines this variation in mycorrhizal growth response, i.e. how much growth benefit a plant has from interacting with a certain fungus, remains unknown. An important first step to understanding the mycorrhizal response is understanding if the genetic organization of AM fungi adapts to different environments and plant hosts, impacting their growth.

Among fungi, AM fungi have relatively large genome sizes (~ 150–750 Mb) and are rich in transposable elements [6]. They form mycelia with a shared cytoplasm containing many nuclei, ranging from hundreds in spores to millions in grown mycelial networks [5, 6, 11]. Such coenocytic hyphae generally lack cross-walls and nuclei can flow freely from the hyphae into the spores as they form and grow [12, 13]. As a result, spores contain hundreds of nuclei and there is no known single nucleus stage that generates the next generation. Although other fungi with multinucleate hyphae and spores are known [14, 15], to our knowledge the extremely large coenocytic nuclei number and apparent lack of a single nucleus stage is unique to AM fungi.

Another confounding aspect about the genetics of AM fungi is that sexual structures have never been observed [6]. Therefore, historically AM fungi were thought to propagate asexually, raising questions about their ability to purge deleterious mutations and to generate genetic variation required for adaptation. One mechanism proposed a large variety of genetically diverse nuclei in fungal individuals, and subsequent selection on individual nuclei [5]. However, the availability of various whole genome sequences from different AM fungi has somewhat challenged this view, revealing much lower intra-organismal genetic variation than previously assumed [16–23]. Furthermore, AM fungi were found to contain a full complement of the core genes required for meiosis [17, 24, 25]. A putative mating-type (MAT) locus, consisting of two HD-like genes, has been identified in *Rhizophagus irregularis,* consistent with a bipolar mating system [20]. Whole genome sequencing together with single nucleus sequencing of various *R. irregularis* isolates revealed that some were in fact monokaryotic (ie. containing genetically very similar nuclei representing one nucleotype with a single MAT allele), while others (such as isolates A4 and A5) appeared to be dikaryotic (ie. two different nucleotypes carrying two distinct MAT alleles). Furthermore, allele frequency analyses indicated a mostly 1:1 ratio of the two nucleotypes in the two dikaryotic strains studied [20]. A recent study by the same group suggested that in dikaryotic strains the ratio of the two nucleotypes may shift in response to host plant identity [26]. Interestingly, recent RAD sequencing of the dikaryote-like C3 isolate, which is closely related to the A4 isolate [27, 28], showed that progeny lines grown on the same host can already vary substantially in the ratio of the two nucleotypes [29]. Similar observations were previously made based on a polymorphic genetic marker [30, 31] and AFLP analyses [7].

To get more detailed insight into the organization of intra-strain genetic variation and especially the impact of different host plants at the genome and transcriptome level, we focused on the putative dikaryote-like *R. irregularis* C3 isolate, because of its presumed high level of intra-genomic variation [27, 28]. By using a combination of culture meta-genome, single spore and single nucleus sequencing as well as RNA sequencing in different host plants we reveal a highly dynamic genomic organization.

## Results

### Characterising intragenomic variation

#### Genome assembly

**Fig. 1:**
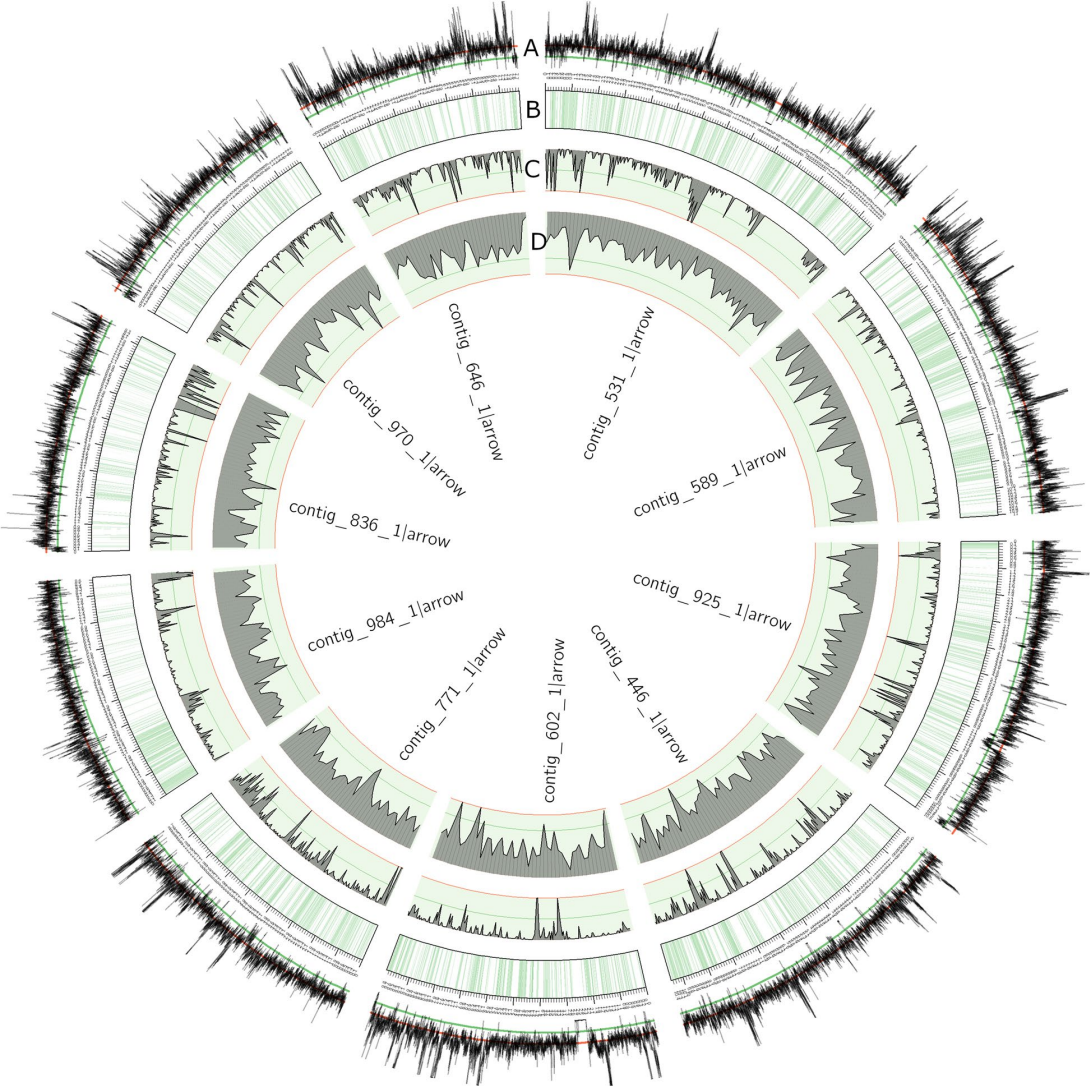
Circos diagram of the 10 largest contigs of the RirC3 assembly, representing 9.9 Mb. **a** Mapping depth of C3 Illumina reads, green line = 50, red line = 100. **b** Physical map of the contigs, with coding regions coloured green. **c** SNP density, green line = 10, red line = 20. **d** Repeat density, green line = 75, red line = 100

The *R. irregularis* C3 isolate was initially chosen because of its reported relative high level of genetic variation, based on RADseq data [28], which in hindsight was overestimated due to lack of an appropriate reference genome [32]. As no reference genome for C3 was yet available wefirst generated a C3 reference assembly, using a combination of PacBio and Illumina sequencing on genomic DNA extracted from a large number of spores and hyphae from axenic *Daucus carota* root cultures, to be able to characterize the genetic variation in this isolate (Fig. 1; Table 1). This resulted in an assembly (RirC3; Genbank BioProject ID PRJNA747641) comprising 1380 contigs and representing a total length of 155 Mbp (Table 1, Fig. 1); a genome size similar to previous estimates for the genome length in *R. irregularis* strains [6]. A representation of the 10 longest contigs covering 9.9 Mb, depicting the distribution of repeats, coding regions and SNP density, is shown in Fig. 1.

**Table 1:** RirC3 genome assembly overview

Total assembly length	155,051,422
Contigs	1380
Gaps	2
GC content	27.94%
Longest contig length	1,364,683
Average contig length	112.356
Contig N50 length	263.815
Contig L50	174
Contig N90 length	63.299
Contig L90	605
Total repeat length	71,617,038
Percentage of genome composed of repeats	46.19%
Total number of biallelic SNPs in the meta genome	121.109
Non-synonymous SNPs	10,677
Stop codon gains	381
Frameshift gains	487
SNP frequency	0,78/kb
SNP density outside repeats	0,59/kb
Number of predicted genes	27,181
PFAM domains	3714
BUSCO (fungi_odb10)	84.3% (639/758)
Complete and single-copy BUSCO genes	82.6% (626/758)
Complete and duplicated BUSCO genes	1.7% (13/758)
Fragmented BUSCO genes	1.2% (9/758)
Missing BUSCO genes	14.5% (110/758)

Repeated regions, including transposable elements, represented 46% of the genome (71 Mb). These repeat regions appear to be randomly distributed over the genome and the majority remain unclassified (Fig. 1). The genome assembly contained 85% of the BUSCO (fungi_odb10) gene set, which is similar to the completeness observed for the high quality *R. irregularis* DAOM197198 genome (Rir17 [19]; Additional File 1 and closely related isolate A4 [20]. BUSCO genes that were not found include FATTY ACID SYNTHASE I and other genes reported to be consistently lost in the *R. irregularis* genome (Additional File 1), correlating with their obligate biotrophic lifestyle [33]. Nearly all BUSCO genes were found in a single copy, indicating a low level of contig duplication from the two nucleotypes. The RirC3 assembly was further annotated using the FunAnnotate pipeline, adapted for fungal genome annotation [34], resulting in 27,181 predicted gene models.

#### 45S rDNA organization

*R. irregularis* DAOM197198 (Rir17) was reported to contain an atypical non-tandemly repeated organization of the 45S rDNA locus, consisting of 10 or 11 copies [19]. Similarly, RirC3 contains only eight 45S rDNA copies that also lack a tandem organization. Four of these copies were located on separate contigs; the other four were found in two pairs, separated over 50 kb apart on separate contigs.

**Fig. 2:**
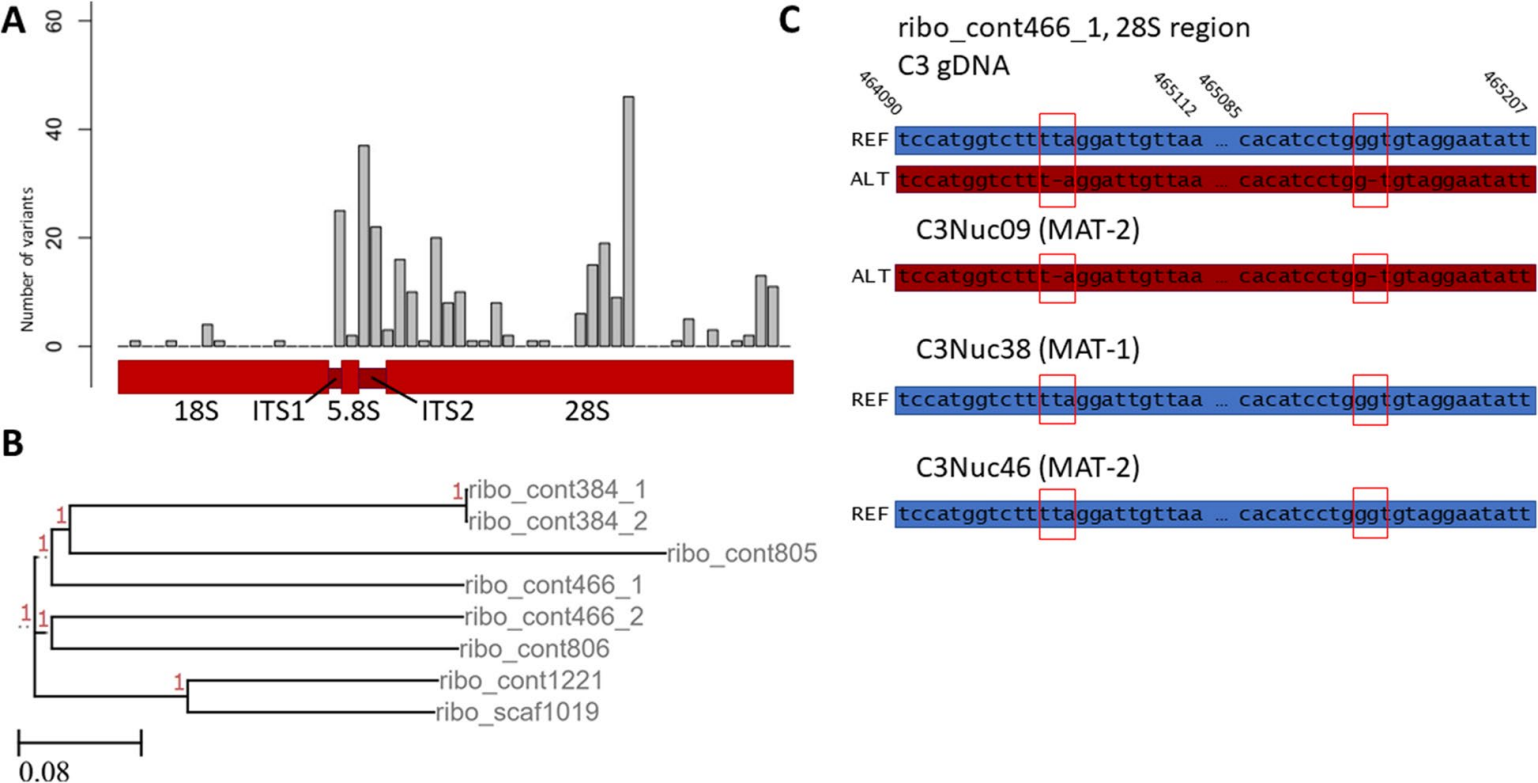
Polymorphisms found in the RirC3 45S rDNA locus. **a** Graph showing the amount of genetic variation among 45S rDNA copies. **b** Phylogenetic representation based on of multiple sequence alignment (1000 bootstraps) of the eight 45S rDNA copies. No copies were identical. The names of the samples correspond to which contig they were found on (e.g. ribo_cont466_1 was the first copy on contig_466 of the RirC3 assembly). Red numbers indicate support values. **c** Example of biallelic SNPs in the rDNA sequence distributed over different nuclei. The reference and alternate alleles for a 28S subregion of ribo_contig466_1 in the C3 gDNA and 3 individually sequenced nuclei (C3Nuc9, C3Nuc38 and C3Nuc46) are shown

Alignment of the sequences of these copies showed significant variation among the different loci, each consisting of 18S rDNA, intergenic spacer region 1 (ITS1), 5.8S rDNA, ITS2, and 28S rDNAs (Fig. 2a,b). When assessing the sequencing depth at these 45S rDNA sequences, we found no increased coverage that would suggest a collapse of assembled sequences as would be expected in the case of many highly conserved copies. Upon analyzing the number of polymorphisms among the 45S rDNA copies, we identified 31 SNP’s within four of the 45S rDNA contigs (Additional File 2). These data support the relative high heterogeneity of *R. irregularis* 45S rDNA copies, which has been suggested to potentially modulate the translational activity of different ribosomes [19]. Single nucleus sequencing (see below) showed that different nuclei indeed encode distinct rDNA alleles (Fig. 2c), confirming the observed heterogeneity in the assembly.

#### Allelic variation

**Fig. 3:**
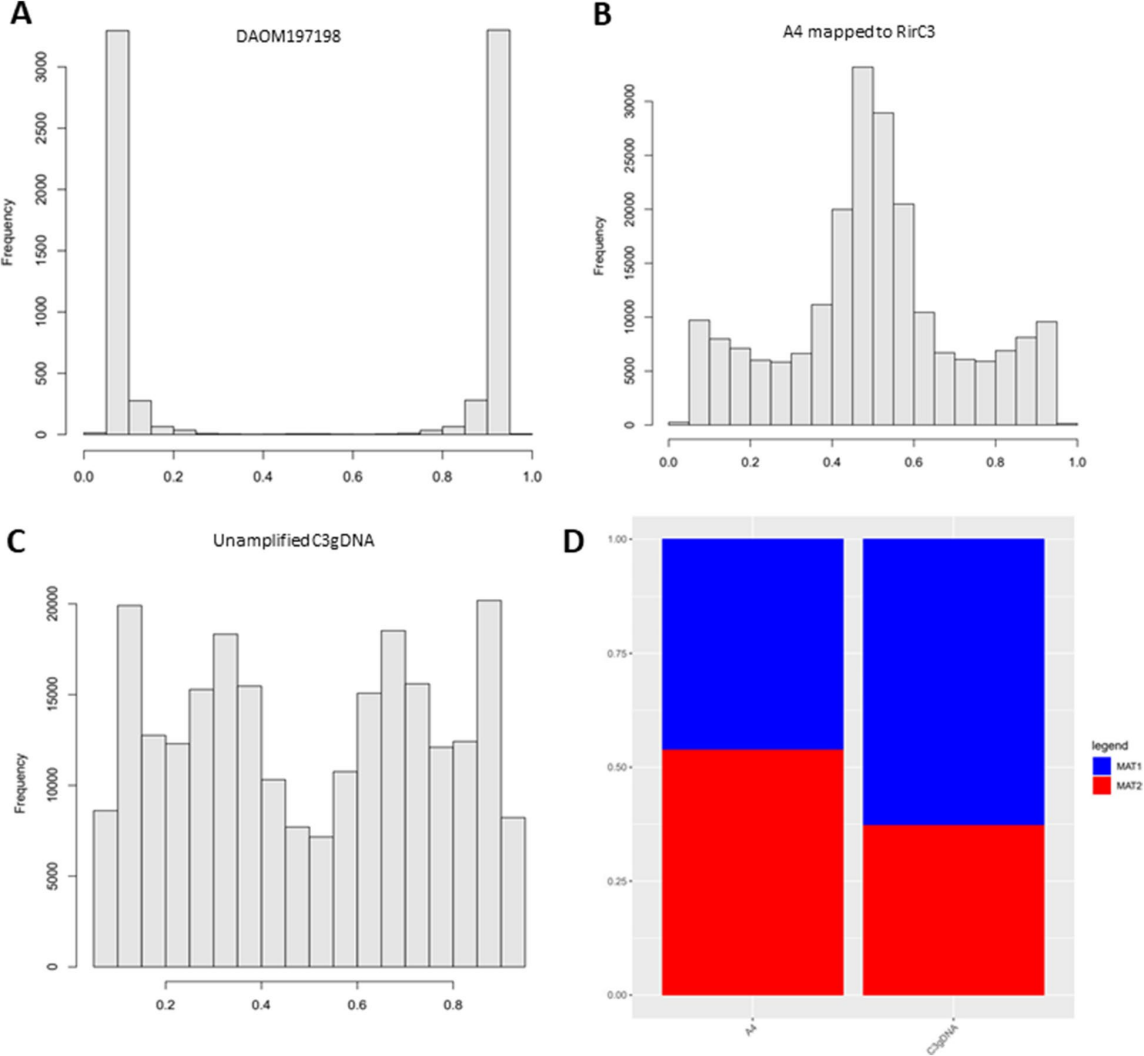
Allele frequencies in different *Rhizophagus irregularis* isolates. Only biallelic SNPs were considered in all samples, where both reference and alternative alleles were observed at least 10 times. **a** DAOM197198, mapped to Rir17 [19] (coverage between 355 and 455). **b** A4 reads [20] mapped to the RirC3 assembly (coverage between 75 and 125). RirC3 was chosen as the reference genome since the mapping rate of A4 Illumina reads was higher with this assembly. **c** C3 Illumina reads mapped to the RirC3 assembly (coverage between 85 and 135). **d** MAT locus proportions based on coverage of the MAT loci in A4 and C3 Illumina reads mapped against the RirC3 assembly

To investigate the genome-wide level of genetic variation, SNP calling was performed using Freebayes based on Illumina sequencing reads from DNA isolated from a large collection of root culture plates, referred to as meta-genome (C3gDNA). SNPs were filtered based on a coverage within the 25th percentile from the average mapping depth (between 80-135x), and at least 10 observations of both alleles. With these settings 121,109 SNPs were found (Additional Files 3 and 4), giving a SNP density of 0.79 SNPs/kb. After removing SNPs that were located inside repetitive regions 0.59 SNPs/kb remained. 10,677 SNPs represented non-synonymous SNPs in the predicted protein coding genes. To compare the observed allele frequencies, similar analyses were performed on previously published Illumina data of DAOM197198 and A4 [19, 20]. Allele frequency distribution analyses confirmed the homokaryotic nature of the DAOM197198 isolate and the reported 50:50 distribution of allelic variations in the A4 isolate (Fig. 3a,b). However, the allele frequency distribution in the C3 meta-genome sample showed two peaks corresponding to 33 and 67% allele frequencies for C3 (Fig. 3c,d). Such an allele frequency distribution is typically found in triploid genomes [35]. The observed 2:1 SNP ratio had a consistent genome-wide distribution, ruling out that this distribution was caused by local aneuploidy.

**Fig. 4:**
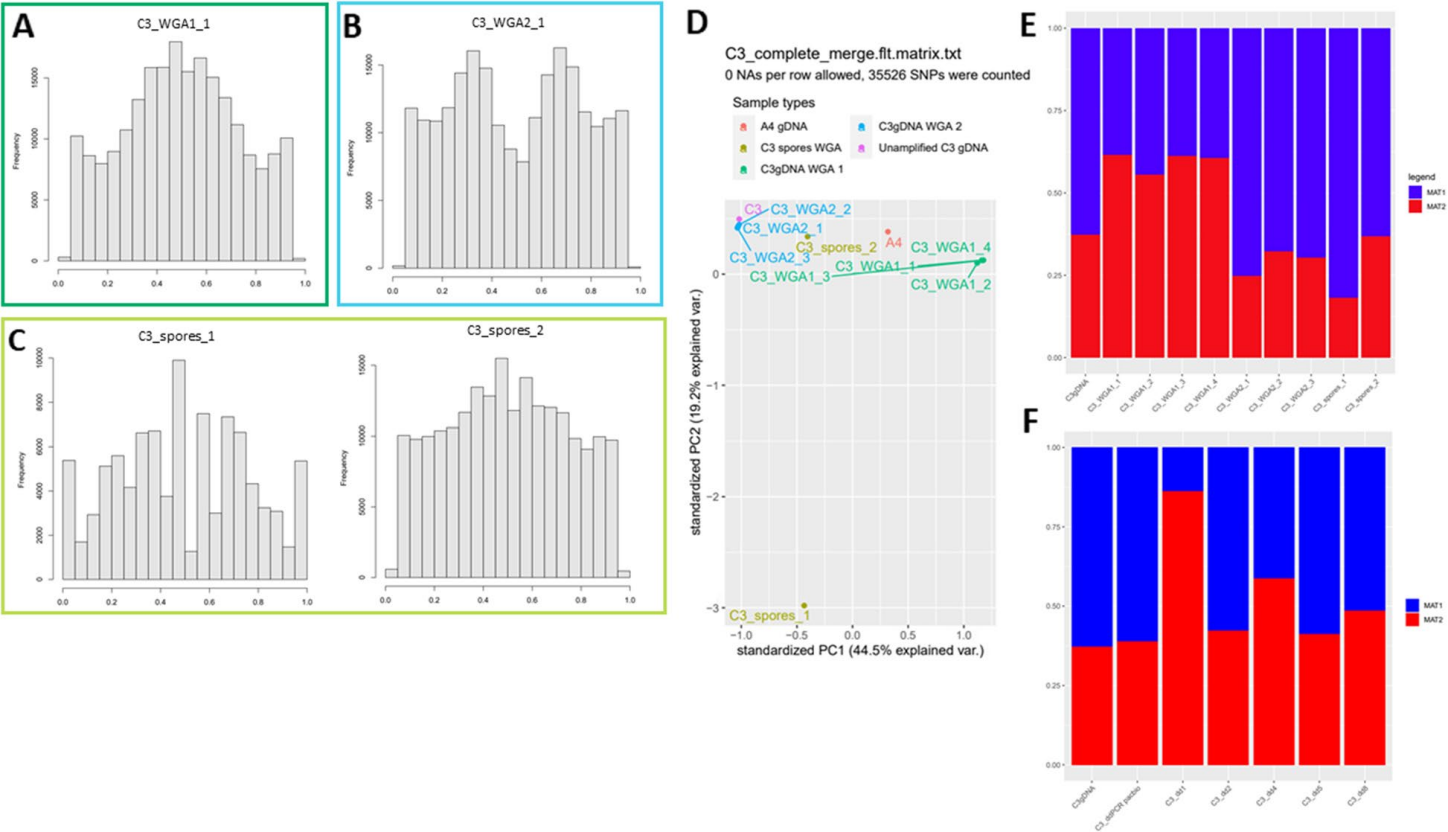
Allele frequencies of different C3 colonies. **a** Allele frequency distribution of C3 genomic DNA that was amplified from the sample used for Illumina sequencing. **b** Allele frequency distribution of amplified C3 genomic DNA from a previously isolated sample. **c** Allele frequencies of two C3 colonies, of which ~ 50 spores and mycelium were isolated and amplified. **d** Principal component analysis of different C3 DNA samples and A4, based on allele frequencies of shared SNPs. **e**: MAT locus proportions based on whole genome Illumina sequencing data of different C3 DNA samples and A4. **f** ddPCR results of other, newly isolated C3 colonies. Note, C3_ddPCR pacbio refers to the WGA amplified version of the DNA shown in lane 1. C3_dd1,2,4,5 and 8 represent unamplified DNA samples for 5 different root culture plates

To determine whether such unequal allele frequencies were consistent between C3 cultures, gDNA of another batch of C3 root culture plates was also sequenced. Whole genome amplification (WGA) was used to generate sufficient DNA before sequencing of this sample (C3_WGA1, Fig. 4a). To rule out any artefacts introduced by WGA, the original C3gDNA sample used for the assembly was also amplified (C3_WGA2) and sequenced (Fig. 4b). To further monitor the reproducibility of the whole genome amplification procedure with respect to SNP frequencies, multiple WGA replicates were included for both meta gDNA samples (meta refers to the use of a large number of spores and mycelium) (Fig. 4c). Principal component analysis (PCA) of allele frequencies showed that the whole genome amplification did not introduce much variation in allele frequencies between technical replicate samples as seen by the tight clustering of these samples (Fig. 4d). This indicated that whole genome amplification did not cause a significant bias in the allele frequencies of the respective samples. However, it also showed that the two meta DNA samples, isolated from different batches of root culture plates, differed in allele frequencies. The C3_WGA2 samples showed allele frequency peaks at ~ 33 and 67% in line with the allele frequency distribution in the unamplified C3gDNA, while the others (C3_WGA1 replicates) showed a rather broad peak around 50% suggestive of a 1:1 nucleotype ratio (Fig. 4a,b, Additional File 9: Fig. S1).

These analyses suggested that different batches can differ in their nucleotype ratio’s, in line with the ddRADseq data from Robbins et al. 2021 [29]. Two additional DNA samples, each from 50 spores collected from two other root culture plates (labelled C3_spores_1 and C3_spores_2), were sequenced after whole genome amplification. These again showed divergent allele frequency distributions based on both genome-wide allele frequencies and MAT allele ratio’s (Fig. 4c,e).

To further investigate the nucleotype ratio’s we searched for the presumed MAT loci in RirC3. We identified two MAT loci identical to the MAT-1 and MAT-2 sequences reported for A4 [20]. Read mapping to these loci showed similar ratios consistent with the genome wide SNP analyses; approximately 1:1 in the C3-WGA2 reads and 2:1 in the C3_WGA1 reads (Fig. 4e). Variable nucleotype frequencies were also observed based on MAT allele ratios determined by ddPCR on multiple unamplified DNA samples collected from different root culture plates (Fig. 4f), further confirming that the observed variation was not caused by the whole genome amplification.

The presence of the same MAT loci and near 100% mapping of the A4 Illumina reads to the RirC3 assembly (Additional File 17: Table S1) confirmed the very close relationship between these two isolates. Both strains were harvested as single spores from different parts of the same field in Switzerland and axenic root cultures using *Daucus carota* as host plant were initiated ~ 20 years ago [36, 37]. Many SNPs, even low frequency SNPs, were found to be conserved between A4 and C3, i.e. being variable sites in both, although not necessarily at similar frequencies (Additional File 4).

In most basidiomycete fungi, despite migration of nuclei, exchange of mitochondria does not occur during hyphal anastomosis. Previous studies suggested that anastomoses between closely related AM fungi could lead to exchange of genetically divergent mitochondria [38]. However, only one mitochondrial parental haplotype was found in derived single spore cultures [39]. This has been suggested to occur through an active segregation mechanism by which one mitochondrial haplotype dominated the other. We observed only a single mitochondrial haplotype in C3. Although several low frequency SNPs were found, their number was much lower compared to the SNP frequencies observed in the genomic DNA (Additional File 5). This indicated that the mitochondrial population in this heterogenic strain is also largely homogeneous.

In summary, the characterization of the intragenomic variation showed that there can be substantial variation in allele frequencies among individual cultures of the same strain. To investigate the reasons behind such variation we next looked for signs of inter-nucleus recombination and the (partial) segregation of nuclei during sporogenesis, as well as the effect of different host plants on the genetic variation.

### Mechanisms behind the observed differences in allele distribution

#### Potential inter-nucleus recombination

To determine to which extent the observed allele ratios in the genome correlated with the two MAT loci, we sequenced 10 individual nuclei and matched allele variants with their respective MAT locus for each nucleus. Individual nuclei were collected using a fluorescence activated cell sorter (FACS) and subsequently whole-genome amplified (WGA) before Illumina sequencing [40]. The MAT locus identity of the individual nuclei was determined by PCR analyses (Additional File 10: Fig. S2).

**Fig. 5:**
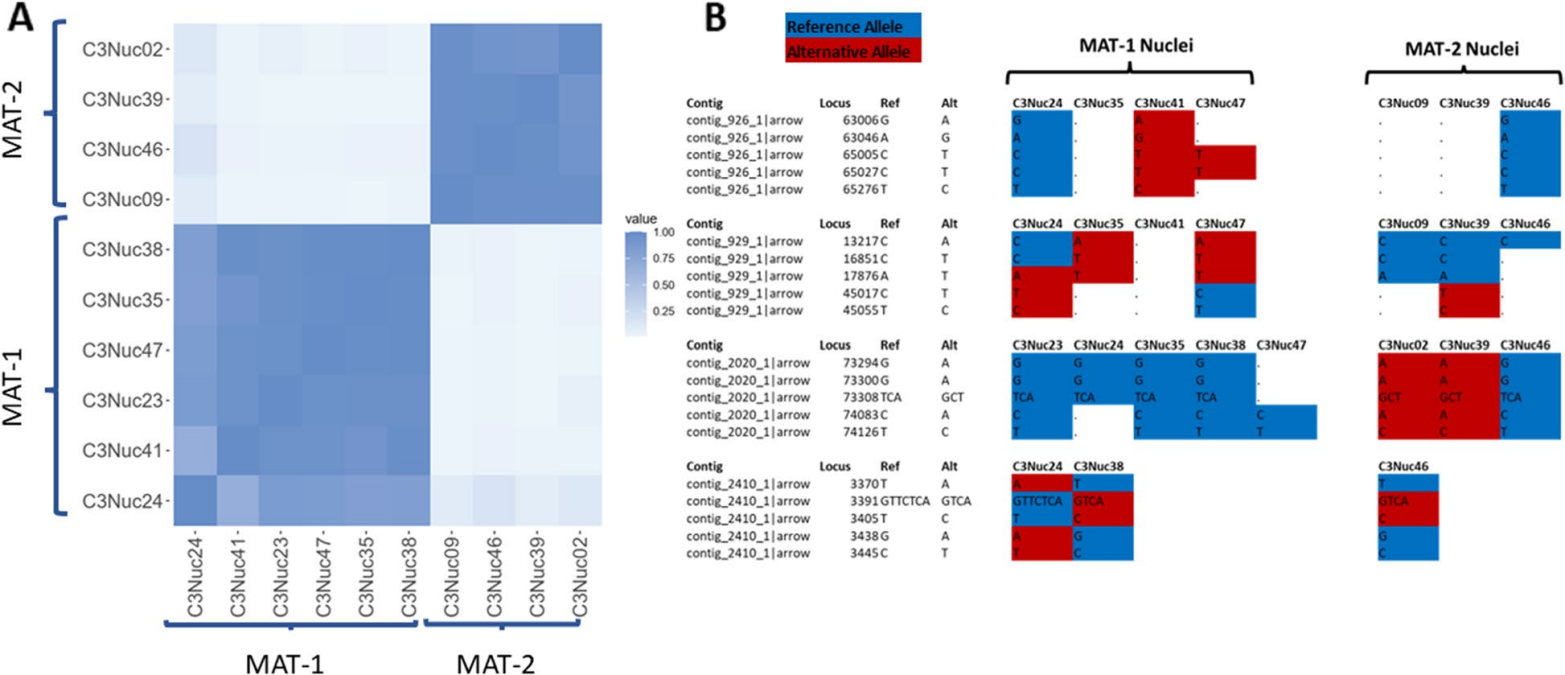
**a.** Similarity plots of C3 nuclei based on single nucleus sequencing data. Color coding indicates level of relatedness among the nuclei. A sharper contrast between the groups means that the nuclei are more different, while patches of differing colors within the groups indicate similarities to nuclei of the other group (meaning the other MAT locus). Nuclei are grouped based on which MAT locus they contain. **b** Examples of genotypes of C3 nuclei not consistent with mating type. Indicated in blue the allele typically found in MAT-1 nuclei (ie. the reference allele called in the assembly), in red the allele typically found in MAT-2 nuclei (alternate allele). The MAT locus identity of the different nuclei is indicated on top. In a true dikaryotic division, all MAT-1 nuclei should have blue alleles, while all MAT-2 nuclei should have red alleles. A complete list of putative recombination sites is given in Additional file 8

Sequencing reads of individual nuclei were mapped against RirC3 and variants were called in parallel using freebayes (Additional File 6). To avoid confounding effects of putative repetitive sequences or potential mapping/assembly artifacts we only considered SNP’s in uniquely mapped reads that were outside genomic regions annotated as repeats. Any loci where two alleles were found in a single nucleus were omitted. Furthermore, SNP’s within 500 bp of these heterozygous SNPs in single nuclei or based on non-paired reads only were omitted. The same analysis was done using Illumina reads of A4 nuclei [21] mapped against RirC3 (Additional File 7). These analyses showed that C3 nuclei clustered together based on MAT locus identity (Fig. 5a; Additional File 11: Fig. S3).

PCA analyses showed that the 6 nuclei containing MAT-1 clustered more closely together with the meta-genomic DNA (C3gDNA), which suggests that this nucleotype mostly contributed the alleles in the assembly (Additional File 11: Fig. S3a). These analyses indicated that most SNPs that were found in the MAT-1 nuclei carried the reference allele called in the assembly, while the MAT-2 nuclei mostly carried the alternative alleles (Fig. 5a; Additional File 6). These analyses further suggested that MAT-2 nuclei are more divergent from each other than the MAT-1 nuclei, which is evident from their clustering less together in the PCA plot (Additional File 11: Fig. S3a). Nuclei with matching MAT loci showed a high level of similarity. Overall, 95% of the SNP’s matched the corresponding/expected MAT locus identity, while 5% of the SNP’s did not; of the 9947 total SNPs, 503 were represented by both alleles among nuclei with the same MAT allele (Additional File 8). After ignoring contigs where only one SNP was found, 408 SNPs remained covering 89 contigs. Blocks of at least 5 consecutive non-matching SNPs (of 244 total SNPs) were found on 25 contigs (examples of some shown in Fig. 5B). Such non-matching sites may point to recombination events between nuclei, as previously suggested for A4 [21, 41, 42].

#### Partial segregation of nuclei during sporogenesis

**Fig. 6:**
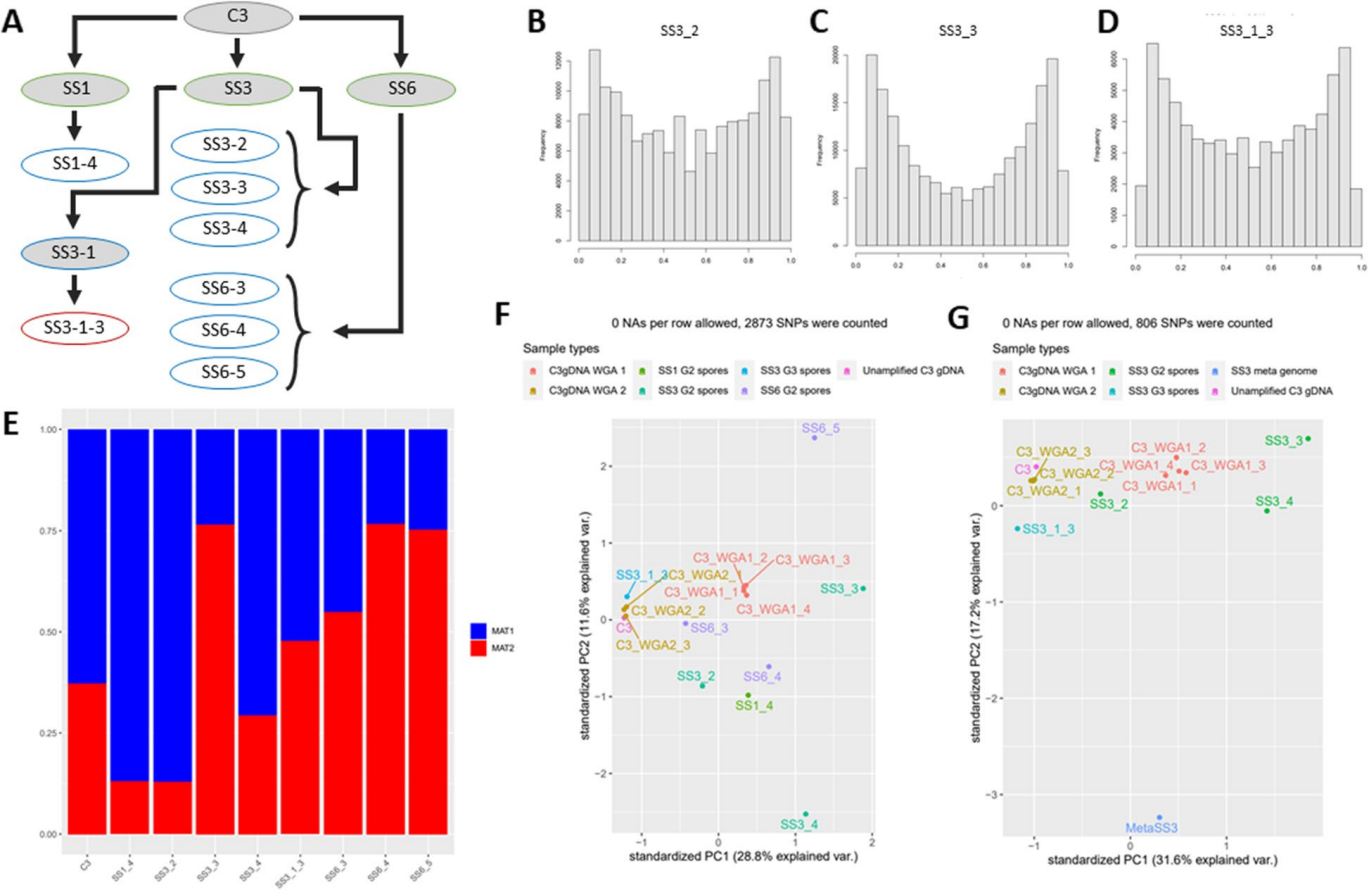
Single spore line variant analysis. **a** Schematic overview of relatedness of all single spore lines. Lines were created by inoculating *D. carota* root cultures with a single C3 spore. Subsequent generations were made by inoculating a new root culture with a single spore derived from the previous single spore line. Generation (G) number is indicated by color: Black = parental C3 colony, green = G1, blue = G2, red = G3. White circles indicate single spores that were amplified and sequenced, grey circles indicate an established colony producing spores. **b-d** Allele frequency distributions of several amplified single spores. **e** MAT loci frequencies of amplified single spores, based on sequencing data. **f** Principal component analysis of single spores based on allele frequencies of shared SNPs. WGA samples were included as additional control samples. **G**: Principal component analysis of single spores derived from SS3, including metagenomic DNA from SS3

Our observed variation in allele frequency distribution between different root culture plates (Fig. 4) raised the suspicion that allele frequencies might be subject to stochastic drift effects. It was previously suggested that varying assortment of genetically different nuclei into newly formed spores can lead to different allele ratios between individual offspring spores [5, 7, 29–31, 43]. This so-called partial segregation of nuclei could bestow individual single spore offspring lines the ability to differentially affect plant performance. For example, it was shown that some single spore lines could increase rice growth by a factor of five compared to other lines from the same starting strain [7]. To test for signals of nuclei segregation at spore formation, three single spore lines (root cultures named SS1, SS3 and SS6) were generated from a single ancestral C3 root culture plate. These single spore lines were re-sequenced together with single spores derived from these lines (Fig. 6a). For example, for SS3 one of its single spores was used to generate a second-round single spore line (SS3–1) and a single spore (SS3–1-3) is derived from it. To obtain sufficient material for sequencing all DNA samples were whole genome amplified.

Patterns of allele frequency distribution varied across single spore lines (Fig. 6b-d) and derived individual spores (Additional File 12: Fig. S4), again indicating that nucleotype composition varies between spores within strains. MAT allele ratio was also variable between these samples, showing that MAT locus based nucleotype composition differs between spores (Fig. 6e). Differential MAT allele proportions were also supported by ddPCR analyses of the MAT alleles in the same samples (Additional File 13: Fig. S5). PCA analyses based on allele frequencies showed that individual spores varied significantly and no signs of convergence of allele frequencies in next generation spores was observed (Fig. 6f,g). Intriguingly, DNA isolated from the single spore line SS1 showed an almost exclusively presence of MAT-2 nuclei, with very little MAT-1 nuclei. Nevertheless, a single spore derived from this line (SS1–4) showed a MAT-1:MAT-2 ratio of 8:1, indicating that individual spores can vary widely in their nucleotype composition. Similar, but less extreme, variation was also observed in second- and third-round progeny spores of SS3 and SS6.

#### Host-dependent differential expression of alleles

**Fig. 7:**
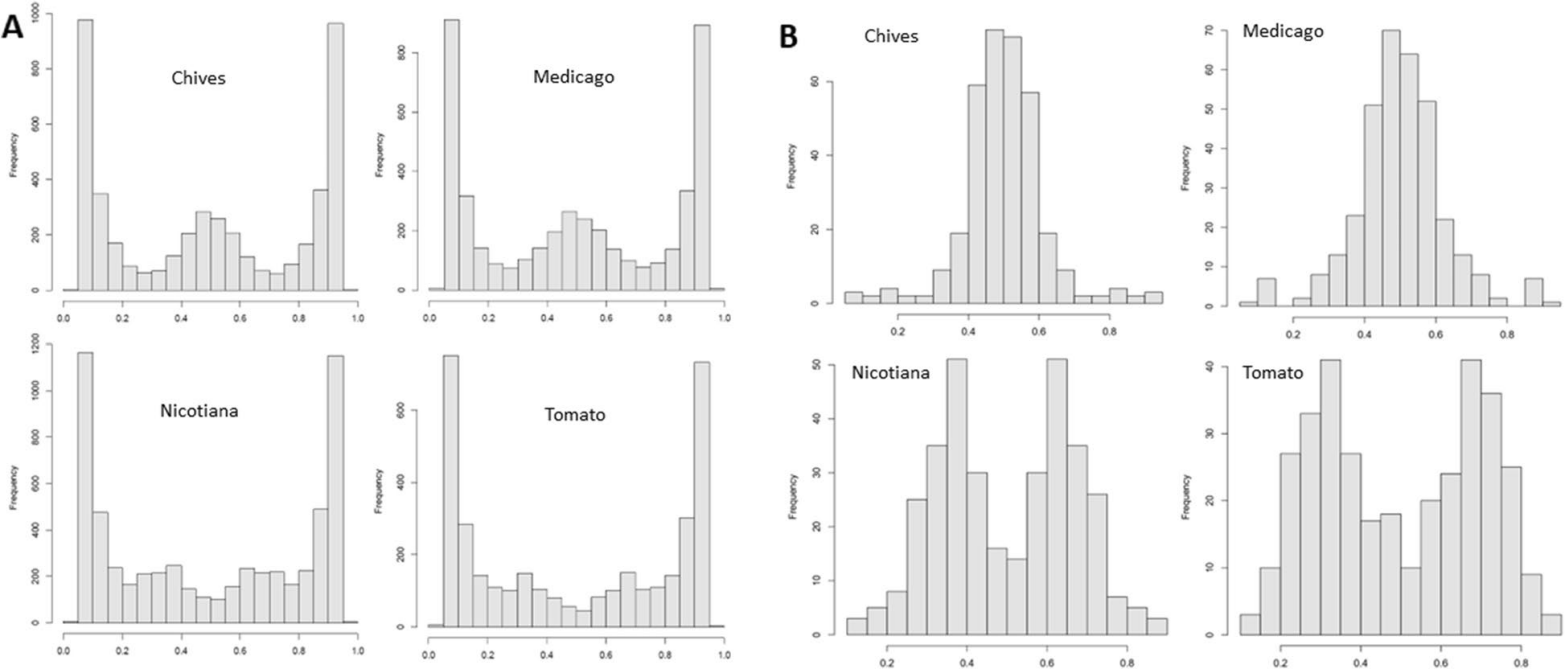
Allele frequency histograms of C3 RNA on different hosts. SNPs were filtered on a minimum sequencing depth of 20 reads, with a of 10 observed reference and alternative alleles. **a** Transcriptome-wide allele frequencies per host. **b** Allele frequencies of SNPs that were sufficiently expressed in all hosts

Variation in nucleotype ratio’s can lead to variation in allele expression [29]. To investigate whether different host plants affect the expression of specific alleles we performed RNAseq analyses of C3 after colonization of *Medicago truncatula* (Medicago), *Nicotiana benthamiana* (Nicotiana), *Allium schoenoprasum* (Chives) and *Solanum lycopersicum* (Tomato) roots. One batch of spore suspension used for inoculation of the different plants was prepared from a separate host, *D. carota* root culture plates. Strikingly, these analyses revealed two different allele frequency distributions in the fungal mRNA populations depending on host plant identity. In the colonized Medicago and Chive roots the C3 mRNA allele frequencies of biallelic SNPs expressed in all four host species showed a clear peak at 50%, while in after colonizing Nicotiana and Tomato there was no peak at 50%, and slight allele frequency peaks at ~ 33 and 67% were observed (Fig. 7a). The same observation in three biological replicates of each plant-fungus combination negates batch effects for each inoculation (Additional File 14: Fig. S6). After filtering on SNPs that were expressed (at least 20 reads) in all four hosts, similar differences in allele frequency distribution were visible, confirming that the allele frequencies of the same genes changed in different hosts (Fig. 7b, Additional File 14: Fig. S6). Since all plants were inoculated with the same spore batch, these data indicated that alleles contributed differently to the mRNA pool when colonizing Medicago and Chives, compared to when colonizing Nicotiana or Tomato. Since alleles are distributed over different (haploid) nuclei the genome-wide shift in allele frequencies it suggests that expression activity varies between nucleotypes.

**Fig. 8:**
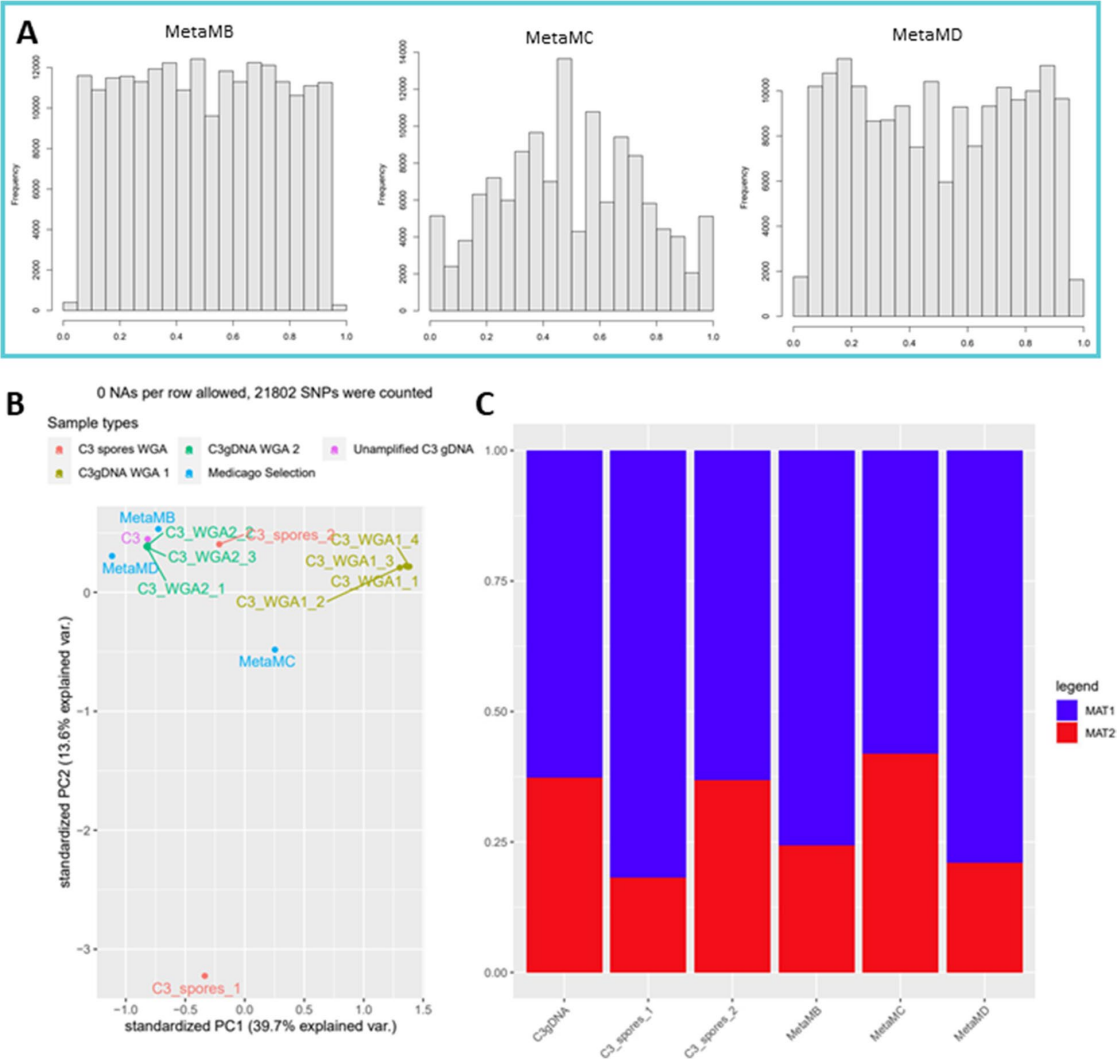
Selection line variant analysis. **a** Allele frequency distributions of three Medicago selection lines. **b** Principal component analysis of C3 and selection lines, based on allele frequencies of shared SNPs. Additional WGA and C3_spores samples were included as additional reference samples. **C**: MAT loci frequency of C3 and selection lines based on read mapping

Unfortunately, we did not have fungal DNA available from the corresponding mycorrhized root samples used for RNAseq. This prevented us from testing whether the observed allele frequency distribution in the RNA reflected already a shift in nucleotype ratios at the DNA level due to the different hosts, as suggested for the A4 isolate [26]. We have previously hypothesized that genetically different nuclei could have different abilities/efficiencies to interact with distinct plant species [44]. For example, certain nuclei could be more adapted to interact with plant species A, whereas other nuclei could be more adapted to interact with plant species B. This could in theory lead to a plant effect on the allele frequencies in the offspring when cultured for a longer time on different plant hosts. To determine whether prolonged growth of C3 on Medicago as a host would lead to a consistent shift in of nucleotype ratio’s, we performed a selection experiment where we subcultured C3 for four rounds, spanning > 2 years, on axenic Medicago root cultures. This resulted in three independent Medicago selection lines that were subsequently sequenced after DNA extraction and whole genome amplification (referred to as MetaMB-D samples in Fig. 8). Unlike the observed 1:1 allele frequencies in the mRNA populations, the prolonged co-culturing of C3 with Medicago did not lead to a consistent shift in nucleotype ratios, based on both genome wide allele frequency distributions and MAT allele ratios (Fig. 8a,b; Additional File 12: Fig. S4). PCA analyses based on allele frequencies did not indicate a closer relatedness of Medicago selection lines compare to different batches of *D. carota* root cultures (Fig. 8b). Furthermore, MAT loci frequencies of these lines showed similar variation (Fig. 8c).

## Discussion

Heterokaryosis is common aspect in fungal biology and is hypothesized to play an important role in the ability of fungi to adapt to a continuously changing environment [45]. In case of the extremely coenocytic AM fungi, it was proposed that changes in nucleotype ratio could be adaptive in the colonization of different host plants [9, 26, 28, 43]. Here, we confirm that the distribution of genetically divergent nuclei in the *R. irregularis* C3 isolate is highly variable, with seemingly random fluctuations of nucleotype ratio. Large variations in allele frequencies were observed between individual (progeny) spores and single spore lines and even between different root culture plates/batches of the same spore line. This is in line with the recent findings of Robbins et al. based on ddRAD sequencing [29]. No consistent effect of host plant identity on the distribution of nucleotypes was observed after 2 years of subculturing on a different host. Interestingly, host identity did have a reproducible effect on allele-specific expression as observed for C3 colonizing four different host plants.

The extent of genetic diversity within AM fungal individuals has been highly debated and the current view is that *R. irregularis* strains are either homokaryotic or dikaryotic [6, 17, 18, 20]. Our analyses show that numerous low frequency polymorphisms are not just mere sequencing artefacts, as suggested by Ropars et al. [20], but real components of the genetic variation within AM individuals that is distributed over different nuclei, in line with Masclaux et al., 2019 [32]. Furthermore, sequencing of multiple replicate amplifications showed that, although some minor fluctuation in allele frequencies was observed, the whole genome amplification procedure did not introduce significant biases. These results show that the term “dikaryotic” does not fully capture the breadth of genetic variation in *R. irregularis* [32], as the coenocytic nature allows for the population of nuclei to accumulate and retain polymorphisms within the nuclear population. This is similar to other fungi, where somatic mutations within an individual lead to polymorphisms that can be maintained through nuclear selection [46]. Intriguingly, multiple low frequency SNPs (occurring between in 10–25% of the reads mapping) were even conserved between the C3 isolate and its presumed clone A4 (Additional File 4). Both strains originated as single spores from different locations in a field in Switzerland and have been individually grown in root cultures for ~ 20 years [36, 37]. Given their very high sequence similarity it therefore seems likely that these isolates once originated from the same parental line(s) in the field. Despite all these years of separation, many SNPs have been maintained within the two isolates, even though their allele ratios can vary substantially. In contrast to the variable nucleotype ratio in C3, A4 was reported to show stable nucleotype ratio among different root cultures and individual spores [20, 26], while in that same study another *R. irregularis* isolate called SL1, the MAT allele ratio was also found not to be stable across spores and subcultures like in C3 [26]. Furthermore, in the very recent work by Cornell et al. (2022) it was found that distinct abiotic factors could affect nucleotype ratio’s. In our case, all root cultures were grown under the same environmental conditions, but still we noticed large variations between spores and batches in C3. What determines this rather different behaviour of nuclei remains to be determined.

Different siblings of the same parental line have been shown to differentially affect plant growth [7]. Recent field trials with different C3 progeny lines in Cassava revealed large differences in cassava growth [47]. Intriguingly, in this work also progeny lines of homokaryotic strains, with much less intra-genomic variation, showed similar strong differential symbiotic effects. This might suggest that in addition to the genetic composition additional factors, such as possible epigenetic effects, contribute to variation in symbiotic performance of lines [47].

C3 contains two main nucleotypes that can be distinguished based on the sequence diversity of two presumed MAT loci. Single nucleus sequencing revealed at least 503 SNP’s in C3 that occurred in different nuclei marked by the same MAT locus. Such SNPs could be the result of somatic mutations and/or point to potential inter-nucleus recombination events between nuclei containing opposing MAT loci. Especially those cases where multiple consecutive SNPs occurred in a single contig and whose allele frequencies in the genome were similar, are strongly suggestive for recombination events. This might be facilitated by the high level of repetitive regions in the genome. The number of putative recombination events was significantly higher than that reported for the closely related A4 isolate [20]. To rule out that this might be due to a different genome assembly, single nuclei data for the A4 strain [20] were analysed with the same settings using the RirC3 assembly (Additional Files 8 and 14). We still found a much lower number of putative recombination events in the A4 data, although the coverage of the A4 single nuclei reads was also lower than those of the C3 nuclei (Additional File 15: Fig. S7) It should be noted that the C3 and A4 data were generated in different labs with potentially different environmental conditions. A variety of external factors such as growing conditions, temperature, starvation or (biotic) stress, as well as intrinsic genetic or epigenetic mechanisms have been linked to recombination rate plasticity [48]. To which extent different recombination rates in *R. irregularis* are conditional remains to be determined.

The allele frequencies varied wildly among progeny spores as well as compared to their parental lines in a seemingly random fashion. Currently, spore formation is thought to represent the most narrow genetic bottleneck in the AM fungal life cycle, where the fewest nuclei (ranging from ~ 60 to thousands) will start a new generation [13]. Single spores that were derived from a previously generated single spore line therefore underwent two genetic bottlenecks compared to the original root culture from which the single spore lines were derived. This could lead to a reduction in genetic variation in subsequent progeny spores. Yet, these second generation single spores were not more similar to each other, but instead varied as much from each other as single spores derived from a different single spore line (Fig. 7). These results illustrate that the genetic composition of a spore is not necessarily representative of the colony that develops from it, in line with data from Ehinger et al., 2012 [30] and Masclaux et al., 2018 [31]. We even found an extreme case where the single spore line 1 contained a large majority of MAT-2 nuclei, with very little MAT-1 nuclei (SS1 in Fig. S4). Although we cannot completely rule out that such an extreme ratio is due to the whole genome amplification, we did see large variation in multiple unamplified samples as well. This may suggest that this line would be on its way to a homokaryotic state, however individual progeny spores derived from SS1 again showed a completely different ratio.

If segregation of nuclei into developing spores would be a truly random process, modelling suggested that this should lead to a loss of diversity and eventual reversion to a homokaryotic state over time [49, 50]. However, the long-term conservation of multiple nucleotypes in C3 indicates that there must be mechanisms that counteract this drift effect. One of these mechanisms may involve continuous nuclear mixing as a result of hyphal fusion/anastomosis, which can occur quite frequently in AM fungi [51–53]. Modelling showed that such mixing could be sufficient to offset the drift effect [55]. Currently, the dynamics of nuclei are not well understood in AMF. Live cell imaging of hyphae found no evidence for synchronized divisions but showed that nuclei can move in “pulses” in a bi-directional manner, seemingly independent form cytoplasmic streaming [54]. How this movement is regulated or coordinated in different parts of the mycelium is not known, but such pulsed movements could ensure the constant mixing of nuclei facilitating the maintenance of the dikaryotic-like status.

Fluctuation in nuclear ratios can also be caused by competition between nuclei [55]. However, also in this case, modelling suggested that it would lead to a loss of diversity in favour of the most dominant nucleotype. Therefore, it was proposed that cooperation, or division of labour, between nuclei could lead to the long-term and stable coexistence of distinct genotypes [56]. Also in other fungi, variation in the ratio of nuclear populations have been observed and suggested to be influenced by nuclear selection [14]. The observed stochastic behaviour of nuclei in C3 would argue against a strong interdependence of nucleotypes. In the absence of varying selection pressures inter-nucleus recombination would be expected to reduce diversity in the long term and lead to the fixation of a single nucleotype. This might explain why most of the current AMF cultures appear to be homokaryotic, since the axenic root cultures represent a more or less homogeneous artificial environment with very little variation [57]. In nature, AM fungi will be exposed to continuously changing environments, such as multiple different host plants and soil characteristics with fluctuations in pH, nutrient sources, water availability or other microbes. All these factors may impose different selection pressures which could favour a heterokaryotic state. It would therefore now be interesting to apply single spore sequencing to spores collected directly from the field to determine the prevalence of dikaryotic-like states, or possibly higher levels of genetic variation.

Upon colonization of different host plants we found that, using the same batch of spores, shifts in allele frequency distribution occurred at the transcriptome level. C3 colonizing Medicago and Chives showed a dominant allele frequency distribution around 50% at the mRNA level for the two MAT nucleotypes, while in the same C3 batch colonizing Nicotiana and Tomato slight allele frequency peaks at ~ 33 and 67% were observed. Observing such reproducible shifts in expressed allele-frequency distributions using the same batch of spores suggests that host-identity not only affects the expression of different genes [58–60] but also different alleles of the same genes. Robbins et al., [29] showed that allele frequencies in the transcriptome of extraradical mycelium mostly resembled the frequency of the two nuclear genotypes in axenic cultures. We cannot completely rule out that the shift in expressed alleles that we observed was caused by a similar host-dependent shift in nucleotype ratio’s, as was shown by [26] for the A4 isolate. However, in our host-selection experiment, which spanned over 2 years, we did not observe reproducible effects on nucleotype ratio’s in C3 upon a host shift from Carrot to Medicagoon (Fig. 8), indicating that if a shift in host-induced nucleotype ratio’s initially occurred it does not seem to be stably maintained. It further remains to be determined whether nuclear ratio’s in the extra-radical mycelium and intra-radical stages are similar.

Nucleotype-specific expression was recently reported for the multinucleate mushroom *Agaricus bisporus*, which contains two to 25 nuclei of two nuclear types per cell. Widespread transcriptome variation was observed between the two nucleotypes in relation to the development of various *A. bisporus* tissues [61]. This was found to be correlated with differential methylation states, suggesting that epigenetic factors may be important regulators of nucleus-specific expression. An additional level of variation may involve the nucleus-specific expression of distinct ribosomal RNA’s. Like DAOM197198, C3 lacked a tandem repeat organization of the 45S rDNA [19]. Eight 45S copies were identified in C3 that showed significant sequence variation and additional polymorphisms were found to be distributed over different nuclei. This may lead to ribosomes with different translational activities in different spores or even different parts of the mycelium [19].

## Conclusions

In conclusion, our analyses show that nuclear behaviour in *Rhizophagus irregularis* can be highly dynamic. The C3 isolate showed inter-nucleus genetic variation and putative recombination, seemingly stochastic (partial) nuclear segregation, root culture batches with varying nucleotype ratios, significant variation in rDNA variants and host-dependent, nucleotype-specific expression. As the combined output of this genetic variation ultimately determines the effect on plant growth promotion [6, 9], further insight into the regulation of such nuclear dynamics will be important to understand their distribution and contribution in ecological settings and to exploit their potential as sustainable biofertilizers in agriculture.

## Methods

### Fungal material

*Rhizophagus irregularis* isolate C3 was originally isolated from a Tänikon, Switzerland as described in [37]. The fungus was propagated on *Agrobacterium rhizogenes*-transformed *Daucus carota* root cultures on M medium [57, 62].

Single spore lines were generated by placing a single C3 spore next to a fresh *D. carota* root culture (initial culture provided by dr. Toby Kiers, University of Amsterdam). Spores were selected from spore clusters from the same source plate, and single spore lines were named after their respective cluster.

Medicago selection lines (MedSel) were made by inoculating *Medicago truncatula* (Jemalong A17) root cultures with ~ 50 C3 spores. When these cultures produced enough spores, these spores moved to fresh *M. truncatula* root cultures to start a new round. Three of these subsequent transfers were made. For DNA sequencing, ~ 50 spores were isolated from the M medium and crushed in 2 μL DNA free mQ water. Total genomic DNA was then amplified using the Repli-G WGA kit (Qiagen).

### DNA isolation for genome assembly

Four square plates, six round plates and four split plates containing fully C3 mycorrhized *D. carota* root cultures were harvested and pooled. Upon harvesting the fungal material, roots were removed from root culture plates with pliers and scalpel, after which the medium was liquidized by adding ½ volumes 100 mM Citrate buffer (40 mM sodium citrate dihydrate, 60 mM citric acid, pH = 6.5) to each volume of M medium and gently shaking at RT for at least 30 minutes. The dissolved medium was then poured into an empty square petri dish, from which the mycelium and spores were collected with a sterile disposable inoculator loop, while taking care to avoid any pieces of the root culture. Collected spores and mycelium were washed in sterile milli-Q water, collected in a 2 mL Eppendorf tube and centrifuged at 5000 rpm. As much water as possible was removed from the tube, after which the sample was weighed and flash-frozen in liquid nitrogen.

Samples were thoroughly (>20x 20s) pulverized with a metal bead in a TissueLyser LT (Qiagen). All materials were kept at minimal temperatures to avoid thawing of the sample. For the isolation of high molecular weight genomic DNA, a protocol from Fauchery et al. [63] was adapted. The lysis buffer was made of five stock solutions (Additional File 18: Table S2) that were combined shortly before the isolation. 1,5 mL of the lysis buffer was added to the frozen fungal material. The sample was mixed by gently shaking until the sample was completely suspended in the buffer. Lysis was performed at 65 °C for 30 minutes, gently shaking every 10 minutes. The lysis was stopped by adding 492 μL 5 M Kac (pH 7.5) and gently inverting. The sample was incubated on ice for 30 minutes and centrifuged at 5000 g at 4C for 20 minutes. The supernatant was transferred to a 15 mL Falcon tube, and cleaned by adding 1 volume chloroform–isoamyl alcohol (24:1 v/v), gently but thoroughly shaking until completely mixed, and pipetting the upper layer to a fresh tube. This step was repeated twice to remove all residual proteins. 10 μL RNAse A (10 mg/ml) was added and the sample was incubated at 37 °C for 1 hour. Next, 20 μL 3 M NaAc (pH = 5.2) was added, the sample was mixed, and then precipitated by adding 1 volume of isopropanol. The sample was incubated at RT for 15 minutes before centrifuging at 4 °C for 30 minutes at max speed. The supernatant was discarded and the pellet was washed with ice cold 70% ethanol. The sample was then dried at RT and resuspended in 55 μL 20 mM Tris-HCl at 65% for 30 minutes. 5 μL of the solution was diluted 4x for quality control, the rest was immediately stored at − 70 °C. Yield was measured by Qubit 2.0 fluorometer via the Qubit dsDNA HS Assay (Life Technologies) and DNA integrity was checked on 0.8% agarose gel. 900 ng of high molecular weight genomic DNA was collected for PacBio sequencing.

### PacBio assembly

PacBio SMRT Sequel2 subreads were generated at GenomeScan B.V. (Leiden, The Netherlands). The subreads were assembled using Flye (2.7.1-b1590) [64] with the following command: flye --pacbio-raw C3_PacBio_subreads.fastq.gz -g 156 m --out-dir C3_assembly --threads 30. Duplicated regions were removed with purge_dups [65]. Genome polishing was performed in two steps: first with the PacBio subreads using Arrow (Pacific Biosciences), then with Illumina reads of C3 using two iterations of Racon (v1.4.13) [66] C3 Illumina reads were produced by sequencing 300 ng of C3 genomic DNA, isolated from the same cultures as the PacBio sample, at NovoGene B.V. (Hong Kong). Genome completeness was assessed with BUSCO [67], using database fungi_db10. Repeats were modelled de novo with RepeatModeler and subsequently masked with RepeatMasker (v. open-4.0.9) [68]. The genome was annotated using Funannotate (v1. 6.0) [34], using predicted gene models and C3 RNAseq reads from C3 grown on multiple hosts (see RNAseq section). The mitochondrial genome was found by blasting RhiirA4 mitochondrial markers [20] against the raw RirC3 assembly (before purge_dups). All markers were found on a single contig covering the entire predicted mitochondrial genome. Ribosomal DNA copies were found by blasting Rir17 rDNA sequences in the RirC3 assembly. Contigs of the assembly were visualized with Circos [69].

### Variant calling

Illumina reads of C3 were mapped against the RirC3 assembly using Hisat2 [70], and sorted with samtools sort. Variant calling was performed on mapped reads using Freebayes (v1.3.2) [71], setting ploidy level to 1 with the pooled-discrete -J option. Only SNPs located outside of repeated regions were counted. Variants were filtered using bcftools filter (v 1.10.2) [72]. SNPs for C3 were filtered on coverage between 85 and 135 (mean coverage 110),and both reference and alterative allele observation of at least 10. Allele frequency distributions were plotted in R (v 4.0.3) using the hist() command. Principal component analysis was performed by merging vcf files with bcftools merge, and creating a dataframe of all allele frequencies in R. Next, only SNPs with coverage in all samples were selected. Principal component analysis was performed using prcomp(df, center = TRUE, scale. = TRUE), and plotted using ggbiplot().

During the preparation of this manuscript another genome assembly was published for the C3 isolate, named CHRIC3 (Robbins et al., 2021). As the CHRIC3 assembly appeared to contain more duplicated genome regions (Additional File 19: Table S3) we continued analyses using our own RirC3 assembly for which the unique read mapping rate was higher. As a comparison, we performed preliminary analyses with both assemblies, showing that either assembly produces similar results regarding the distribution of genetic variation (Additional File 16: Fig. S8).

### Single nuclei isolation and sequencing

Spores of C3 were suspended in 1xPBS buffer (pH 7.4) and crushed with a pestle. Nuclei were selected by fluorescent associated cell sorting (FACS) [40] and whole genome amplified (WGA) through MDA using Phi29 polymerase. An 80x dilution of the reactions was used for genotyping. The remaining reaction mixture was purified using ethanol precipitation and dissolved in 30 μL 10 mM Tris-HCl. Genotyping was done using primers targeting the ITS region (AM1 + NS31) [73, 74] or MAT loci (Forward: ACTATCTGACTTGCTATTGTTGA, Reverse: CAGGGCCTGCATCGGATTA). 10 of the nuclei were sent for Illumina sequencing (NovoGene, Hong Kong). Reads were mapped against RirC3 using HiSat2 [70] with standard settings, and variant calling was performed with freebayes (ploidy = 1). Variants were selected by first intersecting the vcf file with the filtered RirC3 gDNA vcf file, where only SNPs that were found in the whole genome data were selected. SNPs inside repeated regions were ignored. Next loci where any nucleus contained a heterozygous SNP (ie. both alleles were found in a single nucleus) were filtered out as well. Heterozygous SNPs were found using the Awk utility in bash, and were defined as having both alleles at a frequency above 10% (RO/DP > 0.1 || AO/DP > 0.1) in any of the nuclei. Finally, sites in nuclei with a coverage < 10 were ignored. The same selection was done using the A4 single nucleus data [20]. Similarity plots were made in R using ggplot(). Individual nuclear genomes were assembled using Spades [40, 75].

To find potential recombination sites, vcf files were grouped based on MAT identity. Only uniquely mapping reads were considered (samtools view -f 2). SNP’s within 500 bp upstream and downstream of heterozygous loci in and/or based on non-paired reads only, or with a coverage below 10, were ignored. If genotypes of any SNP in the genome were linked to MAT identity of the nuclei, all nuclei sharing a MAT locus should have the same genotype on that SNP. Therefore, any SNP where both the reference and alternative allele were found in nuclei with the same MAT locus, was considered as a non-matching region representing a potential recombination event.

### Single spore amplification and analysis

Single spores were isolated by excising M medium containing spores from root cultures, and subsequently dissolving them in citrate buffer. The spores were thoroughly rinsed with sterile mQ water and collected in 2 μL of mQ in 200 μL PCR strips. Spores were manually crushed using pipette tips of which the tips were briefly melted in an open flame, to create a “pestle”. After crushing, the samples were flash-frozen in liquid nitrogen and incubated at 95 °C for 10 minutes to further lyse the nuclei. WGA was performed using the Repli-G Single Cell kit (Qiagen) following manufacturer’s instructions. The samples were purified by ethanol precipitation and dissolved in 30 μL 10 mM Tris-HCl buffer. Samples were sent for Illumina sequencing in NovoGene (Hong Kong).

### Plant inoculation for RNAseq

#### C3 inoculum

Spores of C3 were released from root cultures by disrupting the root cultures in a blender with 2x volume water and filtering with a 40 μm mesh to capture the spores and mycorrhized root fragments. Spore suspensions were stored at 4 °C.

#### Nicotiana

*Nicotiana benthamiana* seeds were sterilized in 20% bleach solution for 12 minutes, thoroughly washed with sterile water and germinated on water agar with a filter for 72 h at RT, in 16/8 light dark cycle. Pots (9x11x11cm) with 2:1 sterilized clay:silver sand mix were prepared, and ~ 200 C3 spores were added in the middle of the pot, ~ 4 cm below surface. The middle of the surface was covered with a small amount of 1:1 vermiculite/silver sand mix, to act as a more stable soil to plant the small seedling. After germination, seedlings were planted with a fine brush in a 1 mm hole in the vermiculite/silver sand mix. To increase initial growth rate, 2 mL of high phosphate half-strength Hoagland solution [59] (1 mM K2PO4) was added to the seedlings. The pots were covered with plastic foil for the first week to maintain soil humidity, and plants were watered twice a week with adjusted low phosphate half-strength Hoagland solution (50 μM K2PO4). Plants were grown at 25 °C in a 16/8 light dark cycle. Mycorrhized roots were harvested after 6 weeks by gently submerging the pot in water, removing soil and clay from the roots under water and rinsing carefully with tap water.

#### Medicago

*Medicago truncatula* Jemalong A17 seeds were scarified in 96% sulfuric acid for 10 minutes, thoroughly rinsed with water, sterilized with 50% bleach for 10 minutes and washed again with sterilized water. Seeds were then incubated on a water agar plate with filter at 4 °C in the dark, and then incubated at 21 °C in the dark. Pots (9x11x11cm) with 1:1 sterilized clay:silver sand mix were prepared, and ~ 200 C3 spores were added in the middle of the pot, ~ 4 cm below the surface. After germinating, seedlings were planted in the pots. Plants were grown at 21 °C in a 16/8 light dark cycle, and were watered with adjusted half-strength Hoagland solution (20 μM K2PO4). Mycorrhized roots were harvested after 6 weeks and gently washed with tap water.

#### Chives

*Allium schoenoprasum* seeds were soaked in mQ at 4 °C for 8 h, disinfected with 20% bleach for 12 minutes and thoroughly rinsed with sterile water. Seeds were germinated on agar plates with a filter for 48 h at 21 °C (16/8 light dark cycle). Pots (9x11x11cm) with 1:1 sterilized clay:silver sand mix were prepared, and ~ 200 C3 spores were added in the middle of the pot, ~ 4 cm below the surface. After germinating, seedlings were planted in the pots. Plants were grown at 21 °C (16/8 light dark cycle), and were watered with adjusted half-strength Hoagland solution (20 μM K2PO4). Mycorrhized roots were harvested after 6 weeks and gently washed with tap water.

#### Tomato

*Solanum lycopersicum* (MoneyMaker) seeds were soaked in 0.03 M HCl for 6 h and sterilized in 50% bleach for 5 minutes. Seeds were then germinated at 25 °C for 72 h in a 16/8 light dark cycle. Large pots (18x11x11cm) were filled with a 2:1:1 clay:silver sand:vermiculite mix. ~ 200 C3 spores were placed ~ 8 cm below surface. After germination, seedlings were planted and watered with adjusted half-strength Hoagland solution (50 μM K2PO4). Mycorrhized roots were harvested after 8 weeks by gently submerging the pot in water, removing vermiculite and clay from the roots under water and rinsing carefully with tap water.

*Medicago truncatula* (Jemalong A17), *Nicotiana benthamiana*, *Allium schoenoprasum* and *Solanum lycopersicum* (MoneyMaker) were all propagated in-house under greenhouse conditions at Wageningen University (Wageningen, The Netherlands).

### RNA isolation and sequencing

RNA from colonized roots was isolated by flash-freezing colonized roots and destroying the tissue with a cold mortar and pestle. RNA isolation was performed using the RNeasy Mini kit (Qiagen), according to manufacturer’s instructions including an on-column RNAse free DNAse (Qiagen) treatment. Three biological replicates of each treatment were sent for Illumina sequencing (BGI, Denmark). RNAseq reads were mapped to the RirC3 assembly with Hisat2 using the --dta option. Variant calling was performed with Freebayes as described above; only biallelic SNPs found in the genome were used. Variants were filtered based on a minimal coverage in all hosts of 20, with at least 10 observations of both alleles.

### Digital droplet PCR

Digital droplet PCR was performed using 80 ng/μl each of MAT-1 and MAT-2 specific primers [26] and QX200™ ddPCR™ EvaGreen Supermix (BioRad) in a total volume of 20 μl. For unamplified (meta-) samples 2 μl of a 1:10 dilution was used per reaction. For WGA amplified samples, 2 μl of a 1:100 dilution was used as template. The PCR mix was suspended in oil for EvaGreen using the QX200 Droplet Generator (Biorad), following manufacturer’s instructions. PCR was performed for 40 cycles, annealing and elongation at 58 0C. Subsequently, the absolute number of positive droplets was counted using a QX200 Droplet Reader and analysed via QuantaSoft Software (BioRad).

## Supplementary Information

**Additional file 1.** RirC3 BUSCO output and comparison

**Additional file 2.** Additional C3 45S rDNA polymorphisms (.vcf file)

**Additional file 3.** Allele variant (SNP) list based on C3 gDNA reads (.vcf file)

**Additional file 4.** Allele variant (SNP) comparison of C3 and A4 (.vcf file)

**Additional file 5.** C3 mitochondrial DNA variants (.vcf file)

**Additional file 6.** Allele variant (SNP) table of 10 single nuclei from C3 (.vcf file)

**Additional file 7.** Allele variant (SNP) table of 14 single nuclei from A4 (reads from Chen et al., 2018) (.vcf file)

**Additional file 8.** Potential inter-nucleus recombination events in C3 and A4

**Additional file 9.** Fig. S1. Allele frequency distribution of replicate, independently WGA-amplified, C3 gDNA samples; corresponding to main Fig. 4A,B. Two replicates for C3-gDNA2 and 3 replicates for C3-gDNA1 (used for genome assembly)

**Additional file 10.** Fig. S2. PCR analysis of MAT locus identity in C3 single nuclei. The upper band corresponds to MAT-1, the lower band to MAT-2

**Additional file 11.** Fig. S3. Principal component analysis of C3 single nuclei (A) and A4 single nuclei (B) based on allele frequencies when mapped to the RirC3 assembly. The MAT locus identity of the individual nuclei is indicated by color: red = MAT-1, blue = MAT-2

**Additional file 12.** Fig. S4. Allele frequency analysis of (WGA amplified) C3 single spores derived from single spore lines

**Additional file 13.** Fig. S5. MAT ratio based on digital droplet PCR of different root culture batches/lines. C3 pac bio refers to DNA sample C3 gDNA2 used for genome assembly, either unamplified or WGA amplified (Amp1). C3 old refers to the independent DNA sample C3 gDNA1. C3_dd1 and _dd2 refer to WGA amplified DNA from two additional independent C3 carrot root culture batches. C3 meta1 and meta2 refer to DNA extracted and WGA amplified from groups of 50 spores from two different root culture plates. SS1 refers to non-amplified DNA from single spore line 1. MB, MC and MD refer to non-amplified DNA from three Medicago selection lines. SS1_4, SS3_2, SS3_3 and SS6_3 refer to DNA samples from 2nd generation single spore lines

**Additional file 14.** Fig. S6. Similarity plot (heat maps) of A4 nuclei based on single nucleus sequencing data from [21]. Color coding indicates level of relatedness between among the nuclei. A sharper contrast between the groups means that the nuclei are more different, while patches of differing colors within the groups indicate similarities to nuclei of the other group (meaning the other MAT locus). Nuclei are grouped based on which MAT locus they contain

**Additional file 15.** Fig. S7. Allele frequency analysis based on RNAseq data from two additional biological replicate samples of C3 colonizing Chives, Medicago, Nicotiana and Tomato; corresponding to main Fig. 7

**Additional file 16.** Fig. S8. Comparison of the CHRIC3 (Robbins et al., 2021) assemblies on the distribution of genetic variation, showing comparable results as with the RirC3 assembly (this study). A: Mapping depth of C3 Illumina reads against RirC3 (light) and CHRIC3 (dark) assemblies. B: Allele frequencies of SNPs in C3 Illumina reads (C3gDNA) mapped against the CHRIC3 assembly. SNPs were filtered on coverage between 35 and 135x, and both alleles being found at least 10 times. C: Allele frequencies of SNPs in C3 Illumina reads mapped against the RirC3 assembly. Only SNPs were included that were also found when using CHRIC3 as a reference (shown in (B)). D: PCA of C3 nuclei, filtered on SNPs that were commonly found when using both the CHRIC3 and RirC3 assembly. E: Simplot of C3 nuclei (as in fig. 5A), based on SNPs that were commonly found when using both the CHRIC3 and RirC3 assembly

**Additional file 17.** Table S1. Comparison of mapping rate and genome coverage of A4 gDNA and single nuclei data (from [21]), mapped against the RhiiA4 assembly [20] and RirC3. As comparison, the mapping rate of C3 nuclei to RirC3 is included

**Additional file 18.** Table S2. Composition of the lysis buffer mix used for gDNA extraction, used for PacBio sequencing

**Additional file 19.** Table S3. Assembly stats of RirC3 and CHRIC3 assemblies

## Data Availability

The RirC3 assembly with annotation is available at 10.5281/zenodo.7037960. All C3 sequencing data generated in this work are available from Genbank under BioProject ID PRJNA747641 and SRR15179489 - SRR15179534. Sequencing data for A4 were retrieved from BioProject ID PRJNA299206 and PRJNA477348.

## Abbreviations

*AM*: Arbuscular Mycorrhizal
*MAT*: Mating-type
*SNP*: Single nucleotype polymorphism
*WGA*: Whole genome amplification
*ddPCR*: digital droplet PCR
*FACS*: Fluorescence activated cell sorter

## References

[1] Spatafora JW, Chang Y, Benny GL, Lazarus K, Smith ME, Berbee ML, Bonito G, Corradi N, Grigoriev I, Gryganskyi A, James TY (2016). A phylum-level phylogenetic classification of zygomycete fungi based on genome-scale data. Mycologia..

[2] Luginbuehl LH, Oldroyd GE (2017). Understanding the Arbuscule at the heart of Endomycorrhizal symbioses in plants. Curr Biol.

[3] Redecker D, Morton JB, Bruns TD (2000). Ancestral lineages of arbuscular mycorrhizal fungi (Glomales). Mol Phylogenet Evol.

[4] Bruns TD, Corradi N, Redecker D, Taylor JW, Öpik M (2018). Glomeromycotina: what is a species and why should we care?. New Phytol.

[5] Sanders IR, Croll D (2010). Arbuscular mycorrhiza: the challenge to understand the genetics of the fungal partner. Annu Rev Genet.

[6] Kokkoris V, Stefani F, Dalpé Y, Dettman J, Corradi N (2020). Nuclear dynamics in the arbuscular mycorrhizal fungi. Trends Plant Sci.

[7] Angelard C, Colard A, Niculita-Hirzel H, Croll D, Sanders IR (2010). Segregation in a mycorrhizal fungus alters rice growth and symbiosis-specific gene transcription. Curr Biol.

[8] Koch AM, Croll D, Sanders IR (2006). Genetic variability in a population of arbuscular mycorrhizal fungi causes variation in plant growth. Ecol Lett.

[9] Sanders IR, Rodriguez A (2016). Aligning molecular studies of mycorrhizal fungal diversity with ecologically important levels of diversity in ecosystems. ISME J.

[10] Mensah JA, Koch AM, Antunes PM, Kiers ET, Hart M, Bücking H (2015). High functional diversity within species of arbuscular mycorrhizal fungi is associated with differences in phosphate and nitrogen uptake and fungal phosphate metabolism. Mycorrhiza.

[11] Balestrini R, Bianciotto V, Bonfante-Fasolo P (1992). Nuclear architecture and DNA location in two VAM fungi. Mycorrhiza.

[12] Jany JL, Pawlowska TE (2010). Multinucleate spores contribute to evolutionary longevity of asexual glomeromycota. Am Nat.

[13] Marleau J, Dalpé Y, St-Arnaud M, Hijri M (2011). Spore development and nuclear inheritance in arbuscular mycorrhizal fungi. BMC Evol Biol.

[14] Roper M, Simonin A, Hickey PC, Leeder A, Glass NL (2013). Nuclear dynamics in a fungal chimera. Proc Natl Acad Sci.

[15] Roberts SE, Gladfelter AS (2015). Nuclear autonomy in multinucleate fungi. Curr Opin Microbiol.

[16] Martin F, Aerts A, Ahrén D, Brun A, Danchin EG, Duchaussoy F, Gibon J, Kohler A, Lindquist E, Pereda V, Salamov A (2008). The genome of Laccaria bicolor provides insights into mycorrhizal symbiosis. Nature..

[17] Tisserant E, Malbreil M, Kuo A, Kohler A, Symeonidi A, Balestrini R, Charron P, Duensing N (2013). Dit Frey NF, Gianinazzi-Pearson V, Gilbert LB. genome of an arbuscular mycorrhizal fungus provides insight into the oldest plant symbiosis. Proc Natl Acad Sci.

[18] Lin K, Limpens E, Zhang Z, Ivanov S, Saunders DG, Mu D, Pang E, Cao H, Cha H, Lin T, Zhou Q (2014). Single nucleus genome sequencing reveals high similarity among nuclei of an endomycorrhizal fungus. PLoS Genet.

[19] Maeda T, Kobayashi Y, Kameoka H, Okuma N, Takeda N, Yamaguchi K, Bino T, Shigenobu S, Kawaguchi M (2018). Evidence of non-tandemly repeated rDNAs and their intragenomic heterogeneity in Rhizophagus irregularis. Communications biology.

[20] Ropars J, Toro KS, Noel J, Pelin A, Charron P, Farinelli L, Marton T, Krüger M, Fuchs J, Brachmann A, Corradi N (2016). Evidence for the sexual origin of heterokaryosis in arbuscular mycorrhizal fungi. Nat Microbiol.

[21] Chen EC, Mathieu S, Hoffrichter A, Sedzielewska-Toro K, Peart M, Pelin A, Ndikumana S, Ropars J, Dreissig S, Fuchs J, Brachmann A (2018). Single nucleus sequencing reveals evidence of inter-nucleus recombination in arbuscular mycorrhizal fungi. Elife.

[22] Kobayashi Y, Maeda T, Yamaguchi K, Kameoka H, Tanaka S, Ezawa T, Shigenobu S, Kawaguchi M (2018). The genome of Rhizophagus clarus HR1 reveals a common genetic basis for auxotrophy among arbuscular mycorrhizal fungi. BMC Genomics.

[23] Venice F, Ghignone S, Salvioli di Fossalunga A, Amselem J, Novero M, Xianan X, Sędzielewska Toro K, Morin E, Lipzen A, Grigoriev IV, Henrissat B (2020). At the nexus of three kingdoms: the genome of the mycorrhizal fungus Gigaspora margarita provides insights into plant, endobacterial and fungal interactions. Environ Microbiol.

[24] Halary S, Malik SB, Lildhar L, Slamovits CH, Hijri M, Corradi N (2011). Conserved meiotic machinery in Glomus spp., a putatively ancient asexual fungal lineage. Genome biology and evolution.

[25] Tisserant E, Kohler A, Dozolme-Seddas P, Balestrini R, Benabdellah K, Colard A, Croll D, Da Silva C, Gomez SK, Koul R, Ferrol N (2012). The transcriptome of the arbuscular mycorrhizal fungus Glomus intraradices (DAOM 197198) reveals functional tradeoffs in an obligate symbiont. New Phytol.

[26] Kokkoris V, Chagnon PL, Yildirir G, Clarke K, Goh D, MacLean AM, Dettman J, Stefani F, Corradi N (2021). Host identity influences nuclear dynamics in arbuscular mycorrhizal fungi. Curr Biol.

[27] Croll D, Wille L, Gamper HA, Mathimaran N, Lammers PJ, Corradi N, Sanders IR (2008). Genetic diversity and host plant preferences revealed by simple sequence repeat and mitochondrial markers in a population of the arbuscular mycorrhizal fungus Glomus intraradices. New Phytol.

[28] Wyss T, Masclaux FG, Rosikiewicz P, Pagni M, Sanders IR (2016). Population genomics reveals that within-fungus polymorphism is common and maintained in populations of the mycorrhizal fungus Rhizophagus irregularis. The ISME journal.

[29] Robbins C, Cruz Corella J, Aletti C, Seiler R, Mateus ID, Lee SJ, Masclaux FG, Sanders IR (2021). Generation of disproportionate nuclear genotype proportions in Rhizophagus irregularis progeny causes allelic imbalance in gene transcription. New Phytol.

[30] Ehinger MO, Croll D, Koch AM, Sanders IR (2012). Significant genetic and phenotypic changes arising from clonal growth of a single spore of an arbuscular mycorrhizal fungus over multiple generations. New Phytol.

[31] Masclaux FG, Wyss T, Mateus-Gonzalez ID, Aletti C, Sanders IR (2018). Variation in allele frequencies at the bg112 locus reveals unequal inheritance of nuclei in a dikaryotic isolate of the fungus Rhizophagus irregularis. Mycorrhiza..

[32] Masclaux FG, Wyss T, Pagni M, Rosikiewicz P, Sanders IR (2019). Investigating unexplained genetic variation and its expression in the arbuscular mycorrhizal fungus Rhizophagus irregularis: A comparison of whole genome and RAD sequencing data. PLoS One.

[33] Miyauchi S, Kiss E, Kuo A, Drula E, Kohler A, Sánchez-García M, Morin E, Andreopoulos B, Barry KW, Bonito G, Buée M (2020). Large-scale genome sequencing of mycorrhizal fungi provides insights into the early evolution of symbiotic traits. Nat Commun.

[34] Palmer J, Stajich J. nextgenusfs/funannotate: funannotate v1.6.0. Zenodo. 2019. 10.5281/zenodo.3354704.

[35] Viruel J, Conejero M, Hidalgo O, Pokorny L, Powell RF, Forest F, Kantar MB, Soto Gomez M, Graham SW, Gravendeel B, Wilkin P (2019). A target capture-based method to estimate ploidy from herbarium specimens. Front Plant Sci.

[36] Jansa J, Mozafar A, Anken T, Ruh R, Sanders I, Frossard E (2002). Diversity and structure of AMF communities as affected by tillage in a temperate soil. Mycorrhiza..

[37] Koch AM, Kuhn G, Fontanillas P, Fumagalli L, Goudet J, Sanders IR (2004). High genetic variability and low local diversity in a population of arbuscular mycorrhizal fungi. Proc Natl Acad Sci.

[38] de la Providencia IE, Nadimi M, Beaudet D, Rodriguez Morales G, Hijri M (2013). Detection of a transient mitochondrial DNA heteroplasmy in the progeny of crossed genetically divergent isolates of arbuscular mycorrhizal fungi. New Phytol.

[39] Daubois L, Beaudet D, Hijri M, de la Providencia I (2016). Independent mitochondrial and nuclear exchanges arising in Rhizophagus irregularis crossed-isolates support the presence of a mitochondrial segregation mechanism. BMC Microbiol.

[40] Montoliu-Nerin M, Sánchez-García M, Bergin C, Grabherr M, Ellis B, Kutschera VE, Kierczak M, Johannesson H, Rosling A. Building de novo reference genome assemblies of complex eukaryotic microorganisms from single nuclei. Sci Rep 2020 ;10(1):1–0. 10.1038/s41598-020-58025-3 PMC6987183 31992756

[41] Chen EC, Mathieu S, Sedzielewska-Toro K, Peart M, Pelin A, Ndikumana S, Ropars J, Dreissig S, Fuchs J, Brachmann A, Corradi N (2019). Correction: single nucleus sequencing reveals evidence of inter-nucleus recombination in arbuscular mycorrhizal fungi. Elife..

[42] Auxier B, Bazzicalupo A (2019). Comment on'Single nucleus sequencing reveals evidence of inter-nucleus recombination in arbuscular mycorrhizal fungi'. Elife..

[43] Angelard C, Tanner CJ, Fontanillas P, Niculita-Hirzel H, Masclaux F, Sanders IR (2014). Rapid genotypic change and plasticity in arbuscular mycorrhizal fungi is caused by a host shift and enhanced by segregation. ISME J.

[44] Limpens E, Geurts R (2014). Plant-driven genome selection of arbuscular mycorrhizal fungi. Mol Plant Pathol.

[45] Strom NB, Bushley KE (2016). Two genomes are better than one: history, genetics, and biotechnological applications of fungal heterokaryons. Fungal Biology and Biotechnology.

[46] Grum-Grzhimaylo AA, Bastiaans E, van den Heuvel J, Berenguer Millanes C, Debets AJM, Aanen DK. Somatic deficiency causes reproductive parasitism in a fungus. Nat Commun 202. 12(1):783. 10.1038/s41467-021-21050-5. 10.1038/s41467-021-21050-5 PMC7862218 33542245

[47] Ceballos I, Mateus ID, Peña R, Peña-Quemba DC, Robbins C, Ordoñez YM, et al. Using variation in arbuscular mycorrhizal fungi to drive the productivity of the food security crop cassava. BioXRiv 2019. 10.1101/830547.

[48] Stapley J, Feulner PG, Johnston SE, Santure AW, Smadja CM (2017). Variation in recombination frequency and distribution across eukaryotes: patterns and processes. Philosophical Transactions of the Royal Society B: Biological Sciences.

[49] Bever JD, Wang M (2005). Hyphal fusion and multigenomic structure. Nature..

[50] Bever JD, Kang HJ, Kaonongbua W, Wang M (2008). Genomic organization and mechanisms of inheritance in arbuscular mycorrhizal fungi: contrasting the evidence and implications of current theories.

[51] Giovannetti M, Azzolini D, Citernesi AS (1999). Anastomosis formation and nuclear and protoplasmic exchange in arbuscular mycorrhizal fungi. Appl Environ Microbiol.

[52] Giovannetti M, Fortuna P, Citernesi AS, Morini S, Nuti MP (2001). The occurrence of anastomosis formation and nuclear exchange in intact arbuscular mycorrhizal networks. New Phytol.

[53] De La Providencia IE, De Souza FA, Fernández F, Delmas NS, Declerck S (2005). Arbuscular mycorrhizal fungi reveal distinct patterns of anastomosis formation and hyphal healing mechanisms between different phylogenic groups. New Phytol.

[54] Bago B, Zipfel W, Williams RM, Piché Y (1999). Nuclei of symbiotic arbuscular mycorrhizal fungi as revealed by in vivo two-photon microscopy. Protoplasma..

[55] Rayner AD (1991). The challenge of the individualistic mycelium. Mycologia..

[56] Kokkoris V, Hart M (2019). In vitro propagation of arbuscular mycorrhizal fungi may drive fungal evolution. Front Microbiol.

[57] Bécard G, Fortin JA (1988). Early events of vesicular–arbuscular mycorrhiza formation on Ri T-DNA transformed roots. New Phytol.

[58] Kamel L, Tang N, Malbreil M, San Clemente H, Le Marquer M, Roux C, et al. The comparison of expressed candidate secreted proteins from two arbuscular mycorrhizal fungi unravels common and specific molecular tools to invade different host plants. Front. Plant Sci. 8:1–18. 10.3389/fpls.2017.00124 PMC5293756 28223991

[59] Zeng T, Holmer R, Hontelez J, te Lintel-Hekkert B, Marufu L, de Zeeuw T, Wu F, Schijlen E, Bisseling T, Limpens E (2018). Host-and stage-dependent secretome of the arbuscular mycorrhizal fungus Rhizophagus irregularis. Plant J.

[60] Mateus ID, Masclaux FG, Aletti C, Rojas EC, Savary R, Dupuis C, Sanders IR (2019). Dual RNA-seq reveals large-scale non-conserved genotype × genotype-specific genetic reprograming and molecular crosstalk in the mycorrhizal symbiosis. ISME J..

[61] Gehrmann T, Pelkmans JF, Ohm RA, Vos AM, Sonnenberg AS, Baars JJ, Wösten HA, Reinders MJ, Abeel T (2018). Nucleus-specific expression in the multinuclear mushroom-forming fungus Agaricus bisporus reveals different nuclear regulatory programs. Proc Natl Acad Sci.

[62] St-Arnaud M, Hamel C, Vimard B, Caron M, Fortin JA (1996). Enhanced hyphal growth and spore production of the arbuscular mycorrhizal fungus Glomus intraradices in an in vitro system in the absence of host roots. Mycol Res.

[63] Fauchery L, Uroz S, Buée M, Kohler A. Purification of fungal high molecular weight genomic DNA from environmental samples. Methods Mol Biol. 2018;1775:21–35. 10.1007/978-1-4939-7804-5_3 29876806

[64] Kolmogorov M, Yuan J, Lin Y, Pevzner PA (2019). Assembly of long, error-prone reads using repeat graphs. Nat Biotechnol.

[65] Guan D, McCarthy SA, Wood J, Howe K, Wang Y, Durbin R (2020). Identifying and removing haplotypic duplication in primary genome assemblies. Bioinformatics..

[66] Vaser R, Sović I, Nagarajan N, Šikić M (2017). Fast and accurate de novo genome assembly from long uncorrected reads. Genome Res.

[67] Simão FA, Waterhouse RM, Ioannidis P, Kriventseva EV, Zdobnov EM (2015). BUSCO: assessing genome assembly and annotation completeness with single-copy orthologs. Bioinformatics..

[68] Smit AF, Hubley R, Green P. RepeatMasker Open-4.0. 2013–2015. http://www.repeatmasker.org.

[69] Krzywinski M, Schein J, Birol I, Connors J, Gascoyne R, Horsman D, Jones SJ, Marra MA (2009). Circos: an information aesthetic for comparative genomics. Genome Res.

[70] Kim D, Paggi JM, Park C, Bennett C, Salzberg SL (2019). Graph-based genome alignment and genotyping with HISAT2 and HISAT-genotype. Nat Biotechnol.

[71] Garrison E, Marth G. Haplotype-based variant detection from short-read sequencing. arXiv preprint arXiv:1207.3907. 2012.

[72] Li H, Handsaker B, Wysoker A, Fennell T, Ruan J, Homer N, Marth G, Abecasis G, Durbin R (2009). The sequence alignment/map format and SAMtools. Bioinformatics..

[73] Helgason T, Daniell TJ, Husband R, Fitter AH, Young JP (1998). Ploughing up the wood-wide web?. Nature..

[74] Simon L, Lalonde M, Bruns TD (1992). Specific amplification of 18S fungal ribosomal genes from vesicular-arbuscular endomycorrhizal fungi colonizing roots. Appl Environ Microbiol.

[75] Bankevich A, Nurk S, Antipov D, Gurevich AA, Dvorkin M, Kulikov AS, Lesin VM, Nikolenko SI, Pham S, Prjibelski AD, Pyshkin AV (2012). SPAdes: a new genome assembly algorithm and its applications to single-cell sequencing. J Comput Biol.

