# Supplementary figures and images for: Stochastic nuclear organization and host-dependent allele contribution in Rhizophagus irregularis
Source: BMC Genomics. 2023 Jan 28;24:53. doi: 10.1186/s12864-023-09126-6 (PMC9883914; doi:10.1186/s12864-023-09126-6)

C3\_WGA2\_2

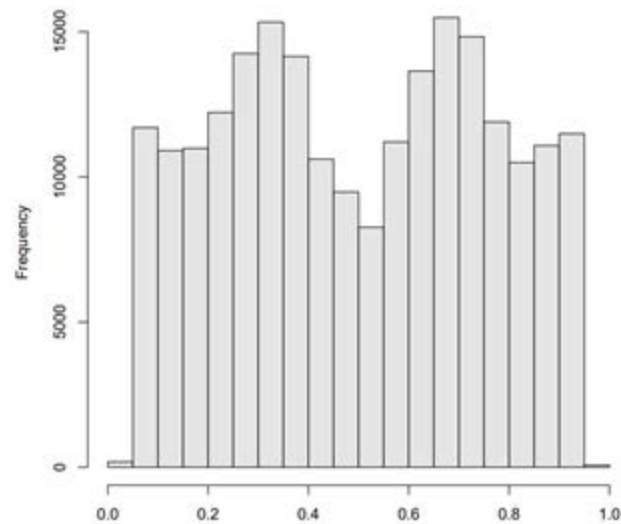

C3\_WGA2\_3

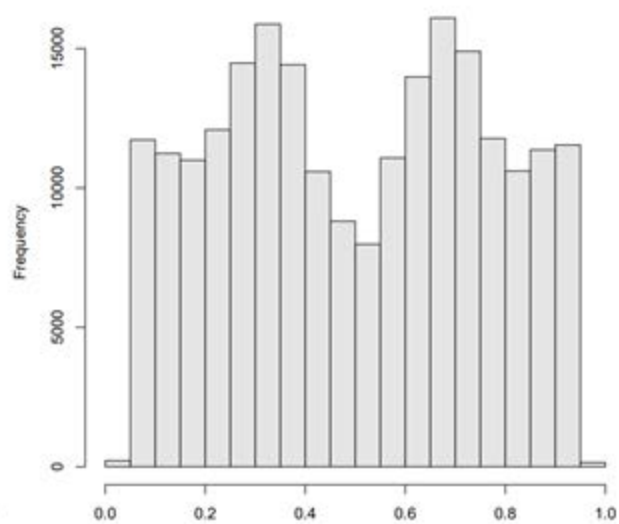

C3\_WGA1\_2

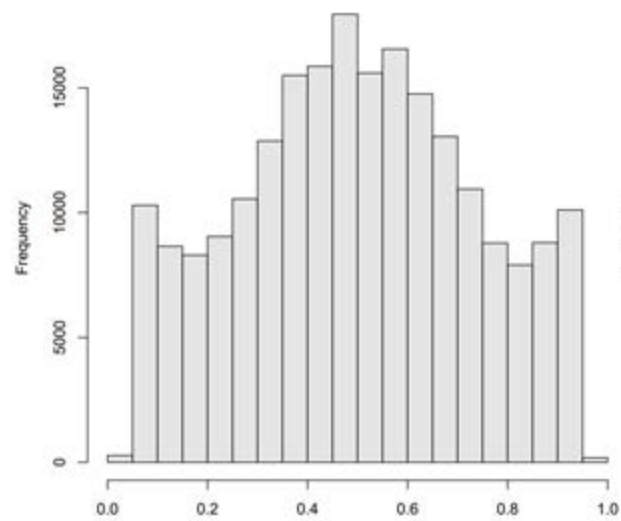

C3\_WGA1\_3

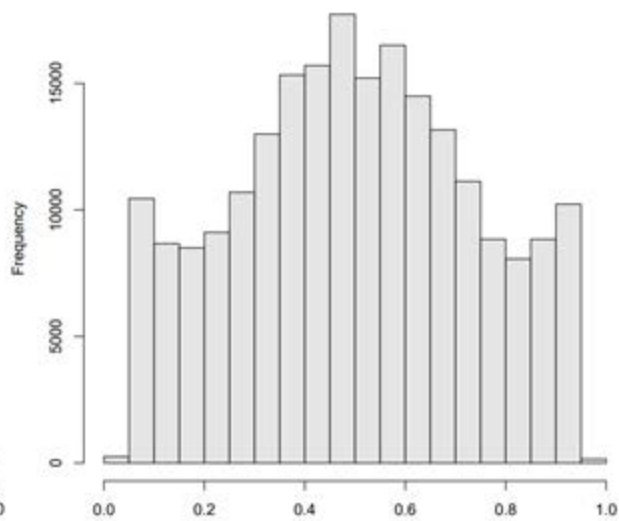

C3\_WGA1\_4

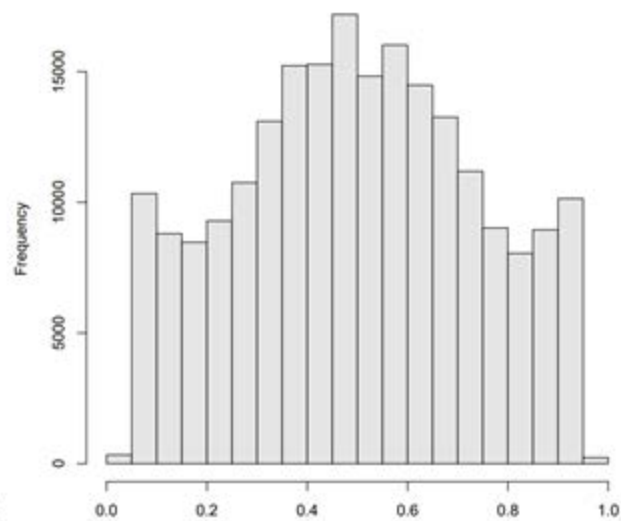

Supplement: Supplementary file 9 — Additional file 9. Fig. S1. Allele frequency distribution of replicate, independently WGA-amplified, C3 gDNA samples; corresponding to main Fig. 4A,B. Two replicates for C3-gDNA2 and 3 replicates for C3-gDNA1 (used for genome assembly) [file 12864_2023_9126_MOESM9_ESM.pdf]

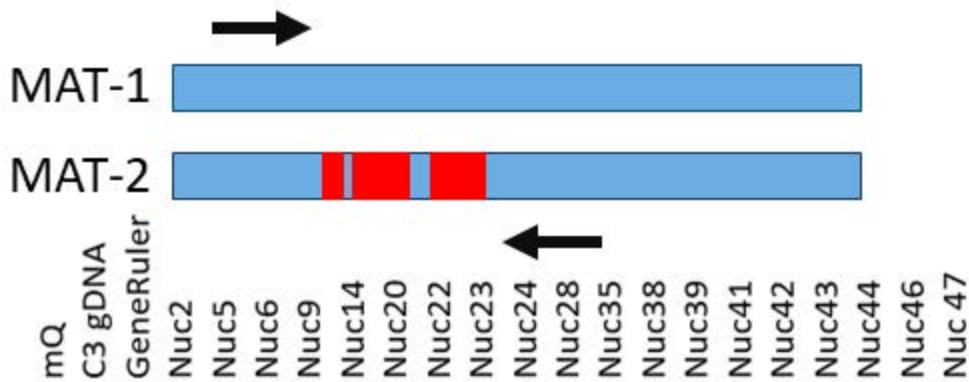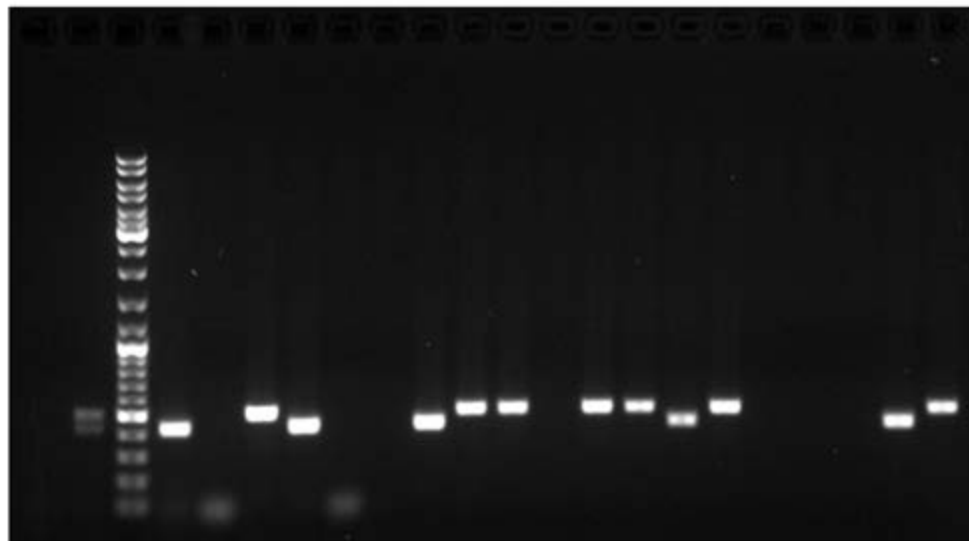

Supplement: Supplementary file 10 — Additional file 10. Fig. S2. PCR analysis of MAT locus identity in C3 single nuclei. The upper band corresponds to MAT-1, the lower band to MAT-2 [file 12864_2023_9126_MOESM10_ESM.pdf]

**A**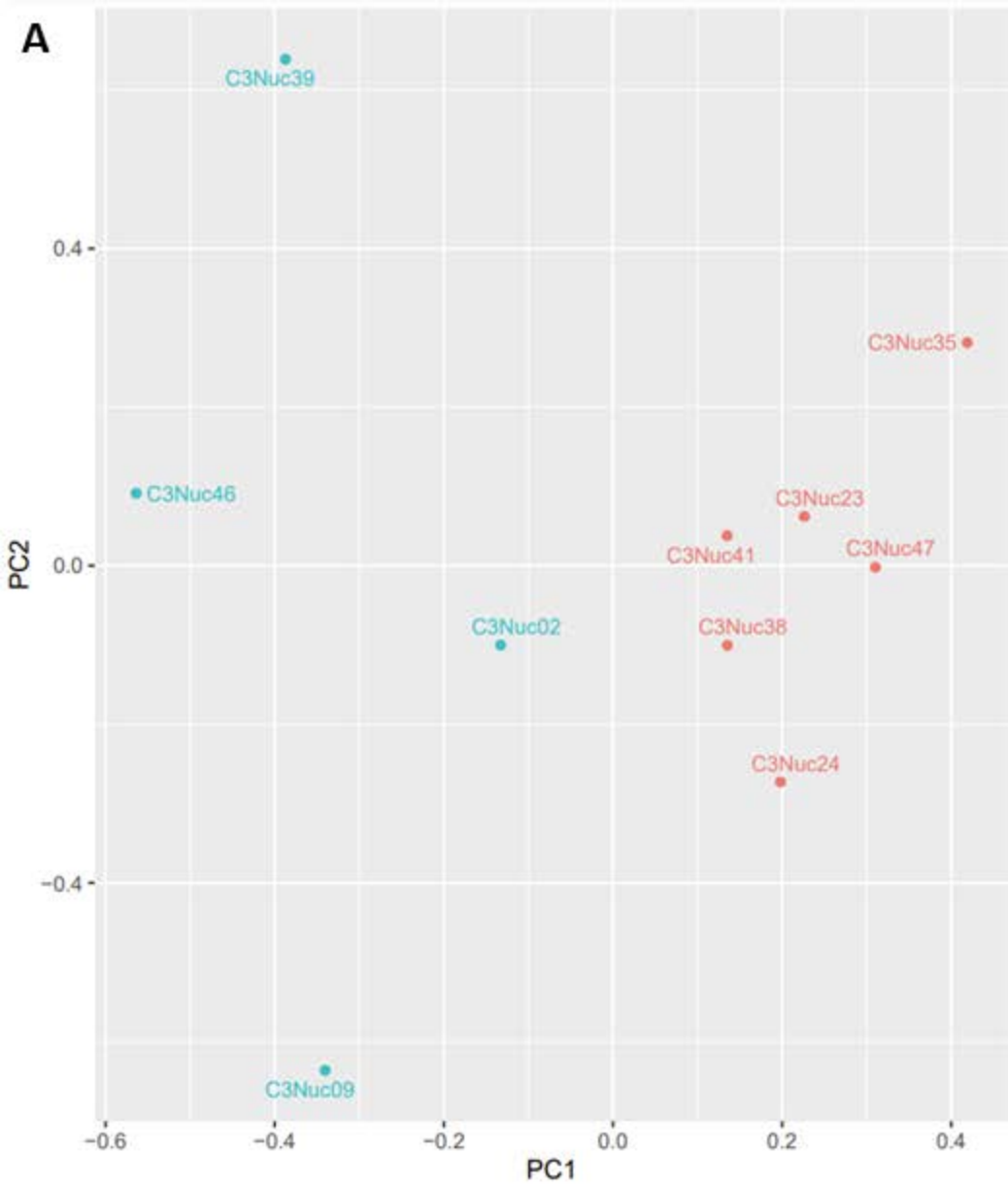**B**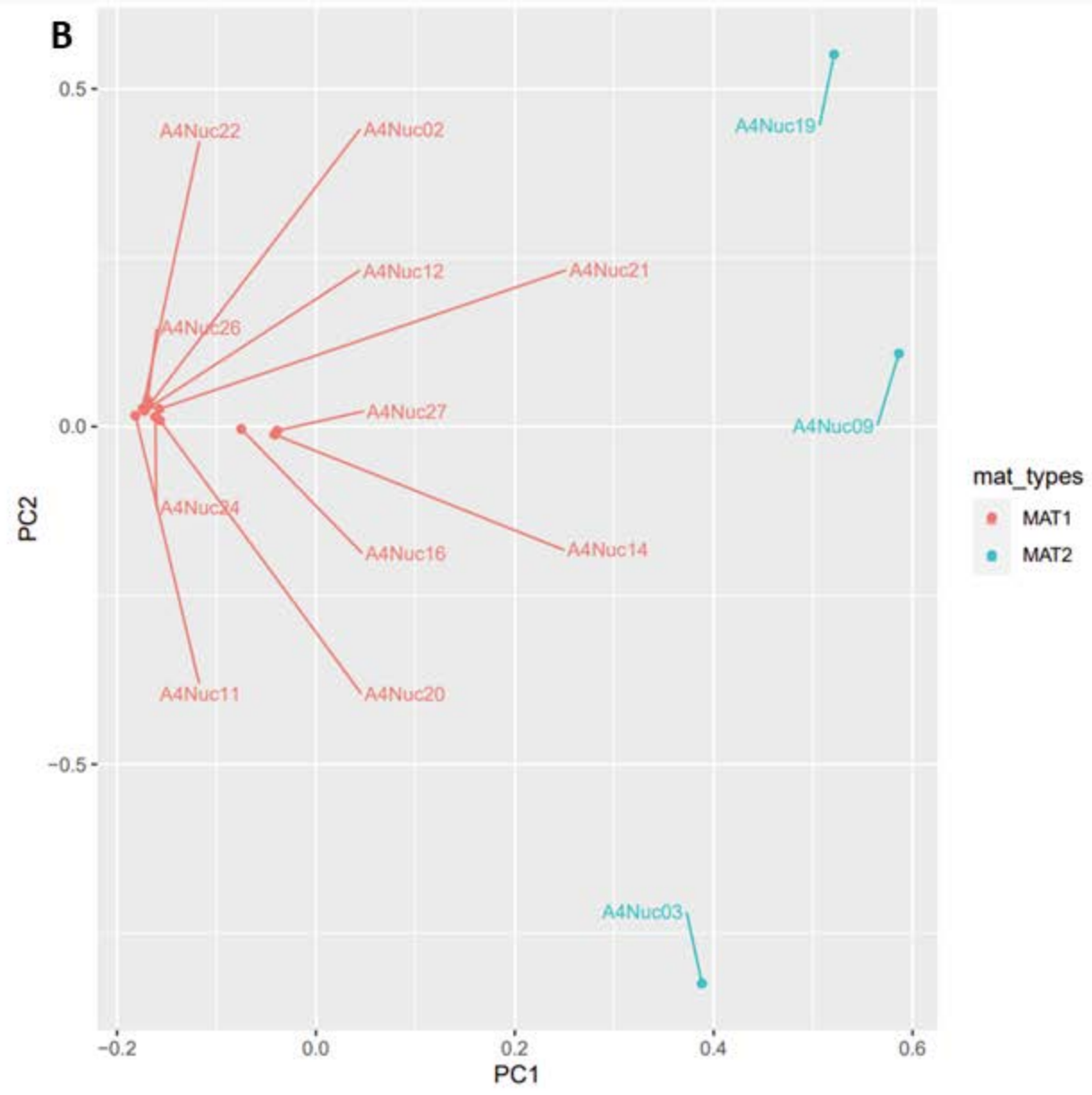

Supplement: Supplementary file 11 — Additional file 11. Fig. S3. Principal component analysis of C3 single nuclei (A) and A4 single nuclei (B) based on allele frequencies when mapped to the RirC3 assembly. The MAT locus identity of the individual nuclei is indicated by color: red = MAT-1, blue = MAT-2 [file 12864_2023_9126_MOESM11_ESM.pdf]

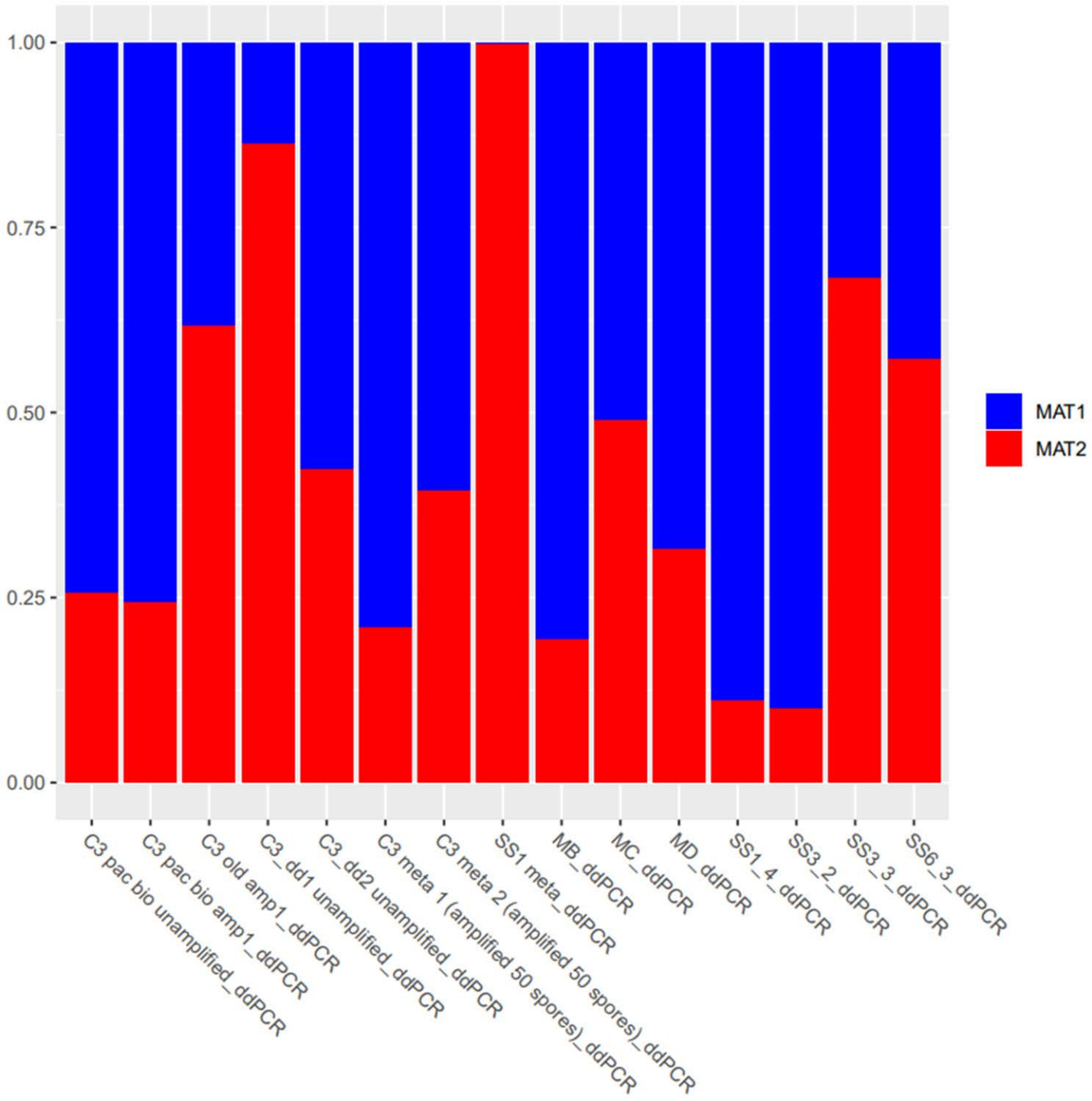

Supplement: Supplementary file 12 — Additional file 12. Fig. S4. Allele frequency analysis of (WGA amplified) C3 single spores derived from single spore lines [file 12864_2023_9126_MOESM12_ESM.pdf]

SS1-4

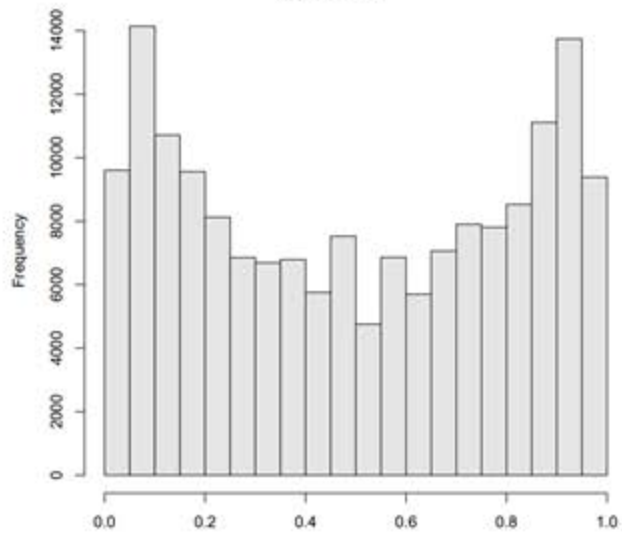

SS3-4

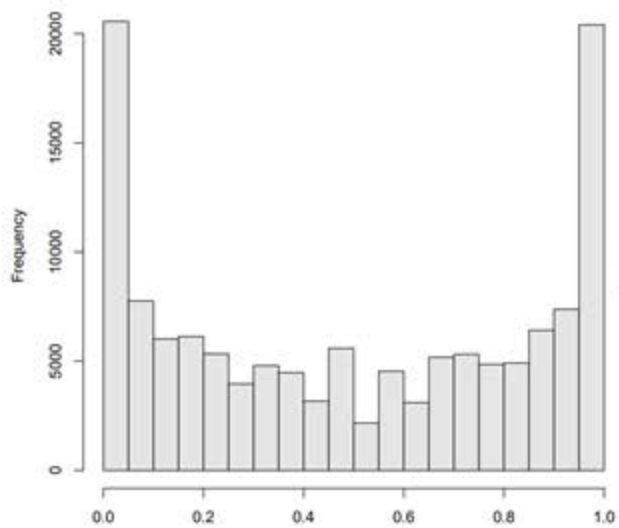

MetaSS3

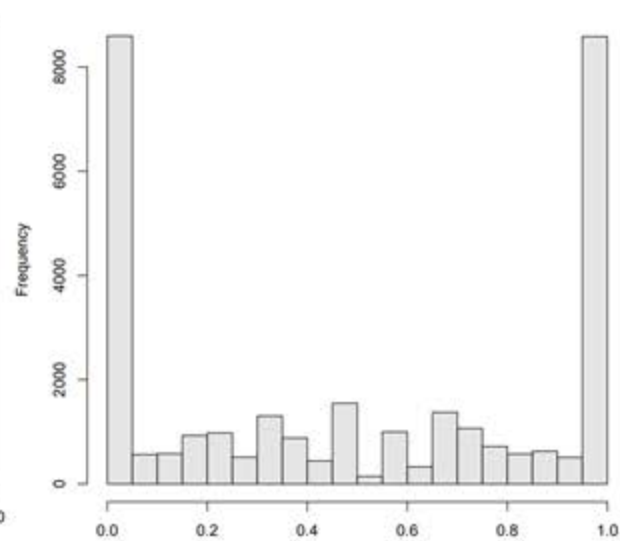

SS6-3

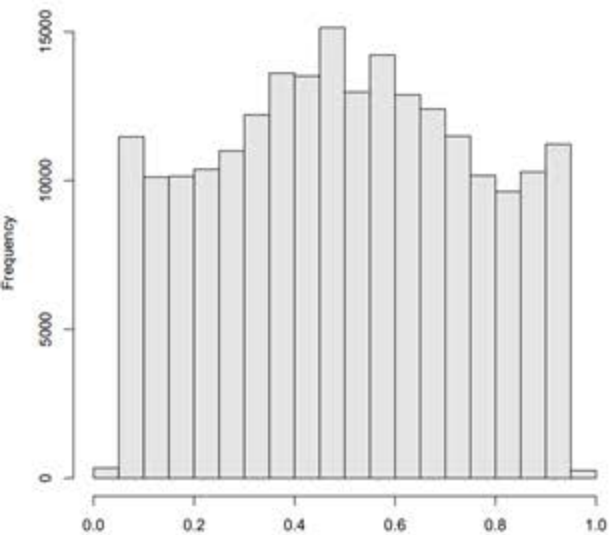

SS6-4

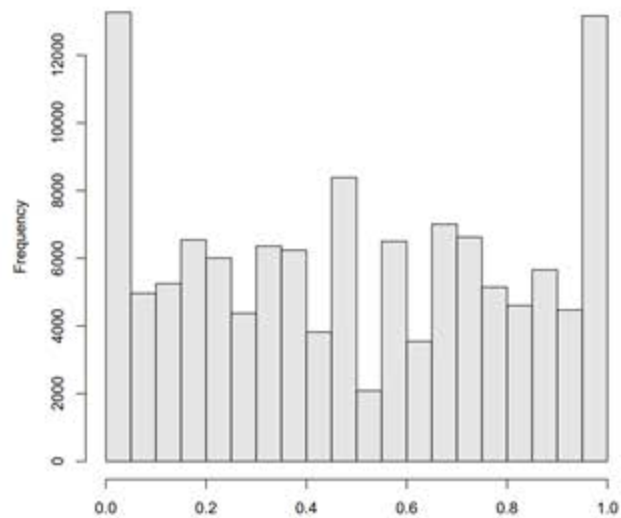

SS6-5

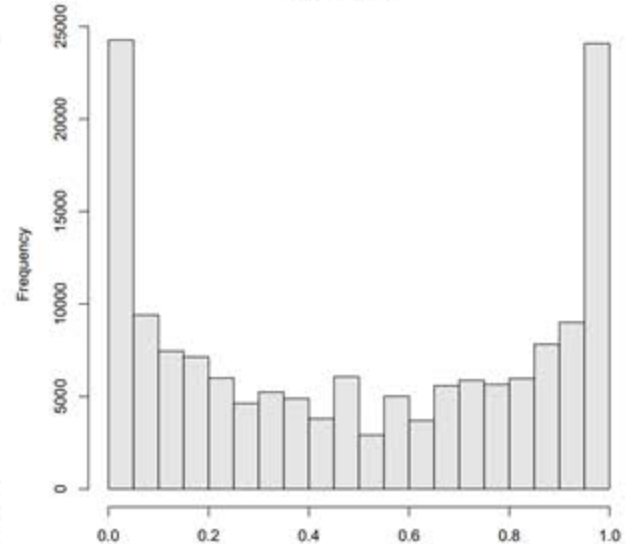

Supplement: Supplementary file 13 — Additional file 13. Fig. S5. MAT ratio based on digital droplet PCR of different root culture batches/lines. C3 pac bio refers to DNA sample C3 gDNA2 used for genome assembly, either unamplified or WGA amplified (Amp1). C3 old refers to the independent DNA sample C3 gDNA1. C3_dd1 and _dd2 refer to WGA amplified DNA from two additional independent C3 carrot root culture batches. C3 meta1 and meta2 refer to DNA extracted and WGA amplified from groups of 50 spores from two different root culture plates. SS1 refers to non-amplified DNA from single spore line 1. MB, MC and MD refer to non-amplified DNA from three Medicago selection lines. SS1_4, SS3_2, SS3_3 and SS6_3 refer to DNA samples from 2nd generation single spore lines [file 12864_2023_9126_MOESM13_ESM.pdf]

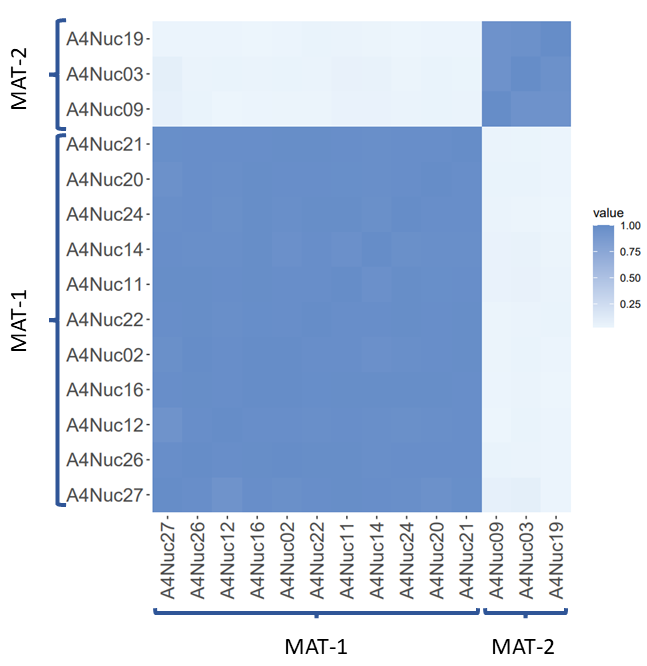

Supplement: Supplementary file 14 — Additional file 14. Fig. S6. Similarity plot (heat maps) of A4 nuclei based on single nucleus sequencing data from [21]. Color coding indicates level of relatedness between among the nuclei. A sharper contrast between the groups means that the nuclei are more different, while patches of differing colors within the groups indicate similarities to nuclei of the other group (meaning the other MAT locus). Nuclei are grouped based on which MAT locus they contain [file 12864_2023_9126_MOESM14_ESM.tif]

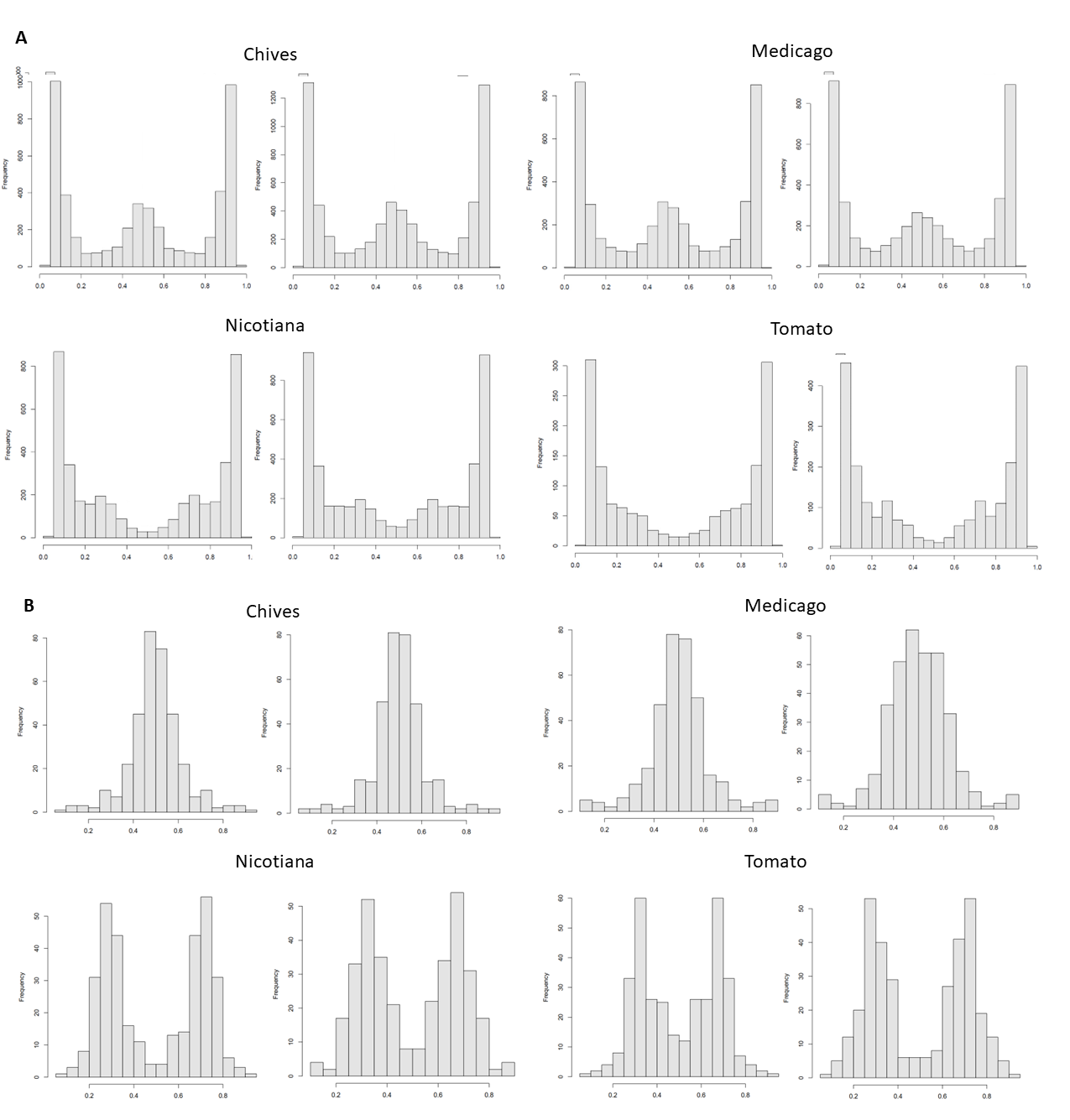

Supplement: Supplementary file 15 — Additional file 15. Fig. S7. Allele frequency analysis based on RNAseq data from two additional biological replicate samples of C3 colonizing Chives, Medicago, Nicotiana and Tomato; corresponding to main Fig. 7 [file 12864_2023_9126_MOESM15_ESM.tif]

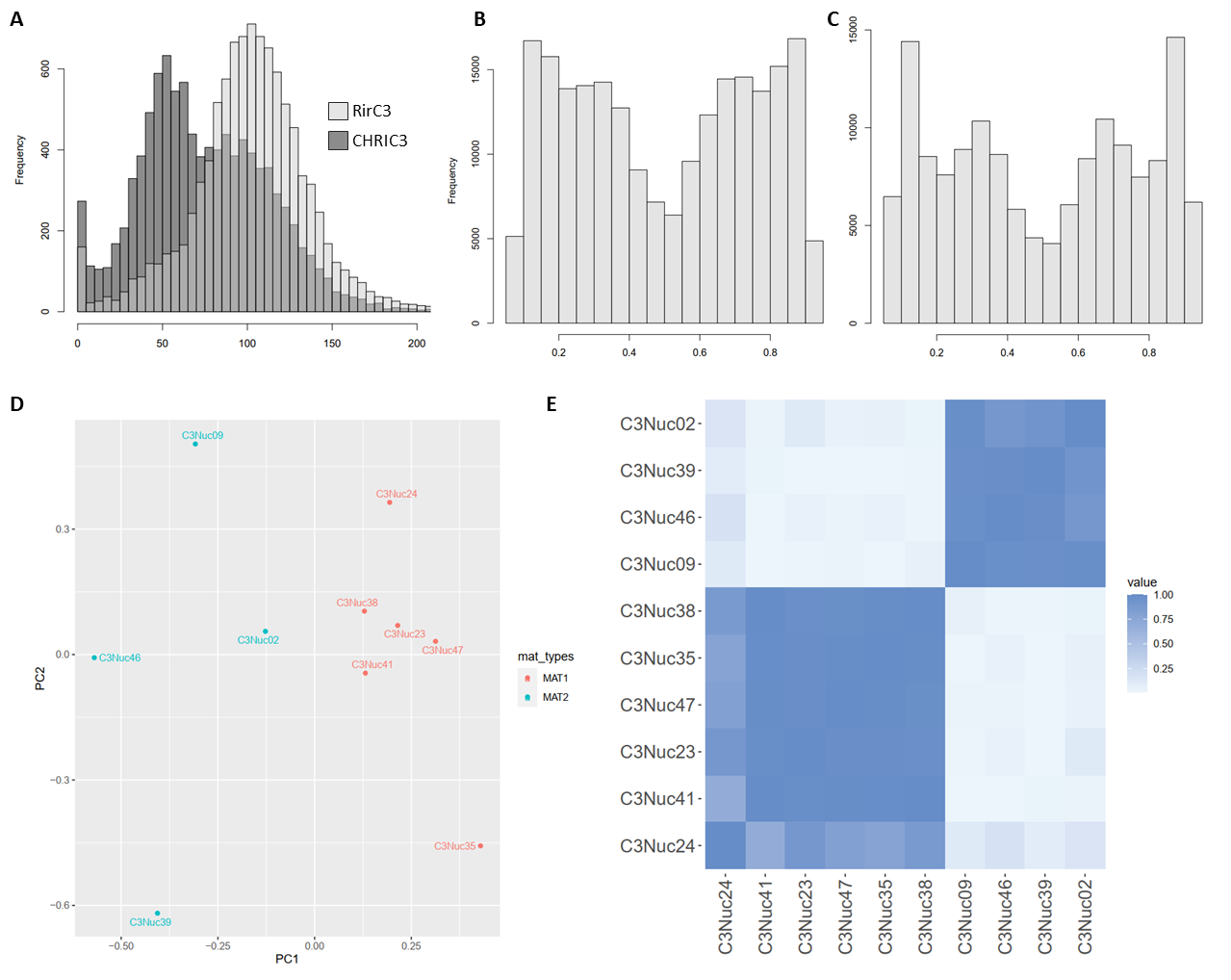

Supplement: Supplementary file 16 — Additional file 16. Fig. S8. Comparison of the CHRIC3 (Robbins et al., 2021) assemblies on the distribution of genetic variation, showing comparable results as with the RirC3 assembly (this study). A: Mapping depth of C3 Illumina reads against RirC3 (light) and CHRIC3 (dark) assemblies. B: Allele frequencies of SNPs in C3 Illumina reads (C3gDNA) mapped against the CHRIC3 assembly. SNPs were filtered on coverage between 35 and 135x, and both alleles being found at least 10 times. C: Allele frequencies of SNPs in C3 Illumina reads mapped against the RirC3 assembly. Only SNPs were included that were also found when using CHRIC3 as a reference (shown in (B)). D: PCA of C3 nuclei, filtered on SNPs that were commonly found when using both the CHRIC3 and RirC3 assembly. E: Simplot of C3 nuclei (as in fig. 5A), based on SNPs that were commonly found when using both the CHRIC3 and RirC3 assembly [file 12864_2023_9126_MOESM16_ESM.tif]
