# Supplementary material for: Stochastic nuclear organization and host-dependent allele contribution in Rhizophagus irregularis
Source: BMC Genomics. 2023 Jan 28;24:53. doi: 10.1186/s12864-023-09126-6 (PMC9883914; doi:10.1186/s12864-023-09126-6)
Supplement: Supplementary file 17 — Additional file 17. Table S1. Comparison of mapping rate and genome coverage of A4 gDNA and single nuclei data (from [21]), mapped against the RhiiA4 assembly [20] and RirC3. As comparison, the mapping rate of C3 nuclei to RirC3 is included [file 12864_2023_9126_MOESM17_ESM.docx]

Table S1

A4 mapping rate and genome coverage

|  | Mapping rate (%) | | Genome Coverage (%) | |
| --- | --- | --- | --- | --- |
|  | To RirC3 | To RhiirA4 | To RirC3 | To RhiirA4 |
| A4 gDNA | 79,05 | 65,98 | 98,66 | 95,13 |
| A4Nuc02 | 70,72 | 62,65 | 22,25 | 18,71 |
| A4Nuc03 | 66,61 | 61,54 | 15,97 | 13,61 |
| A4Nuc09 | 67,6 | 62,68 | 22,14 | 18,86 |
| A4Nuc11 | 69,4 | 60,78 | 20,65 | 17,03 |
| A4Nuc12 | 70,84 | 63,76 | 19,73 | 16,60 |
| A4Nuc14 | 66,45 | 60,83 | 6,79 | 5,75 |
| A4Nuc16 | 66,87 | 60,65 | 10,53 | 9,02 |
| A4Nuc19 | 67,04 | 61,76 | 21,03 | 17,78 |
| A4Nuc20 | 67,68 | 61,39 | 25,67 | 21,24 |
| A4Nuc21 | 65,47 | 59,28 | 20,11 | 16,77 |
| A4Nuc22 | 68,84 | 62,09 | 20,77 | 17,18 |
| A4Nuc24 | 70,38 | 61,15 | 19,78 | 16,24 |
| A4Nuc26 | 66,34 | 59,32 | 20,69 | 17,17 |
| A4Nuc27 | 67,29 | 59,4 | 6,12 | 4,95 |
