# Supplementary material for: Stochastic nuclear organization and host-dependent allele contribution in Rhizophagus irregularis
Source: BMC Genomics. 2023 Jan 28;24:53. doi: 10.1186/s12864-023-09126-6 (PMC9883914; doi:10.1186/s12864-023-09126-6)
Supplement: Supplementary file 18 — Additional file 18. Table S2. Composition of the lysis buffer mix used for gDNA extraction, used for PacBio sequencing [file 12864_2023_9126_MOESM18_ESM.docx]

Table S2:

Lysis Buffer mixture

| **Solution** | **Ingredients** | **Amount in final lysis buffer** |
| --- | --- | --- |
| A | 0.35M sorbitol, 0.1M Tris-HCl pH 9, 5mM EDTA | 6.5mL |
| B | 0.2M Tris-HCl pH 9, 2M NaCl, 2% CTAB | 6.5mL (pre-heated at 65C) |
| C | 5% Sarkosyl | 2.6mL |
| D | 20mg/mL Proteinase K | 125µL |
| E | 10% PVP | 1.75mL |
