# Supplementary material for: Stochastic nuclear organization and host-dependent allele contribution in Rhizophagus irregularis
Source: BMC Genomics. 2023 Jan 28;24:53. doi: 10.1186/s12864-023-09126-6 (PMC9883914; doi:10.1186/s12864-023-09126-6)
Supplement: Supplementary file 19 — Additional file 19. Table S3. Assembly stats of RirC3 and CHRIC3 assemblies [file 12864_2023_9126_MOESM19_ESM.docx]

|  | **RirC3** | **CHRIC3** |
| --- | --- | --- |
| **Assembly size** | 155Mbp | 222Mbp |
| **N count** | 200 | 743399 |
| **Gaps** | 2 | 4646 |
| **Illumina read unique mapping rate** | 83.95% | 66.99% |
| **Illumina read multiple mapping rate** | 6.01% | 25.66% |
| **BUSCO (fungi_odb10)** | 84.3% (639/758) | 94.6% (717/758) |
| **Complete and single-copy** | 82.6% (626/758) | 64.1% (486/758) |
| **Complete and duplicated** | 1.7% (13/758) | 30.5% (231/758) |
| **Fragmented** | 1.2% (9/758) | 1.1% (8/758) |
| **Missing** | 14.5% (110/758) | 4.3% (33/758) |
